# Regional anesthesia strategies for proximal humerus fracture surgery: anatomical considerations, diaphragm-sparing techniques, and expert perspectives—a narrative review

**DOI:** 10.1007/s00423-026-03980-0

**Published:** 2026-03-06

**Authors:** Ezio Spasari, Dario Cirillo, Giuseppe Sepolvere, Giorgio Ranieri, Domenico Pietro Santonastaso, Gennaro Terracciano, Roberto de Giovanni, Paolofrancesco Malfi, Andrea Cozzolino, Antonio Coviello

**Affiliations:** 1 Department of Neurosciences, Reproductive and Odontostomatological Sciences, University of Naples “Federico II”, Naples, Italy; 2 Department of Anesthesia, S. Michele Hospital, Maddaloni, 81024 Italy; 3 Complex Operational Unit of Anesthesia and Operating Units, Department of Emergency and Internal Medicine, Isola Tiberina Hospital, Gemelli Isola, Rome, 00186 Italy; 4 Anesthesia and Intensive Care Unit, AUSL Romagna, M. Bufalini Hospital, Cesena, 47521 Italy; 5 Unit of Orthopedics and Traumatology, Department of Public Health, School of Medicine, University of Naples “Federico II”, Naples, Italy; 6 Department of Life Sciences, Health and Health Professions, Link Campus University, Rome, Italy

**Keywords:** Proximal Humerus Fracture, Shoulder Surgery, Regional Anesthesia, Interscalene Block, Diaphragm-Sparing Techniques, Perioperative Care, Review

## Abstract

**Background:**

Proximal Humerus Fractures (PHFs) are increasingly common, particularly in elderly patients with osteoporotic bone. Surgical and anesthetic management significantly influence outcomes, yet evidence guiding optimal strategies remains limited. Regional Anesthesia (RA) offers effective analgesia, opioid sparing, and the potential to avoid general anesthesia in frail or high-risk patients. This narrative review summarizes current knowledge, highlights diaphragm-sparing approaches, and integrates expert clinical insights to provide practical recommendations for anesthesiologists managing PHF surgery.

**Methods:**

A narrative review was conducted using a structured non-systematic search of PubMed, Embase, and Scopus (January 2000–November 2025). Eligible studies included adults undergoing PHFs surgery with RA and reporting clinical outcomes; case reports, purely anatomical studies, non-surgical analgesia, and non-PHF procedures were excluded. Nine studies met inclusion criteria. Additional references provided anatomical, surgical, and anesthetic context. Orthopedic data were reviewed to contextualize anesthetic strategies, and an “Expert Opinion” section integrated multidisciplinary insights.

**Results:**

Among the nine PHF-specific studies, evidence was limited and heterogeneous, focusing mainly on analgesic efficacy, technical feasibility, and respiratory safety rather than long-term outcomes. Interscalene Brachial Plexus Block (ISBPB) provided effective analgesia but was frequently associated with phrenic nerve involvement. Diaphragm-sparing approaches—such as Superior Trunk Block (STB), infraclavicular techniques, and selective suprascapular and axillary nerve blocks—may reduce respiratory impairment while maintaining acceptable analgesia. Only a minority of studies evaluated continuous RA, reporting prolonged postoperative pain control and opioid sparing in small cohorts. Additional literature supported tailoring block selection to fracture pattern, surgical approach, and patient comorbidities. Expert perspectives emphasized pragmatic, patient-centered strategies, particularly for high-risk or frail patients.

**Conclusions:**

RA is an important component of PHFs surgery, although PHF-specific evidence remains limited. Integrating available data, contextual literature, and expert experience supports an individualized approach balancing analgesic efficacy with safety. Diaphragm-sparing and selected continuous techniques may be considered in patients at increased respiratory risk. Further well-designed studies are needed to refine patient selection and evaluate functional and long-term outcomes.

## Background

Proximal humerus fractures (PHFs) account for 4–6% of all fractures and are the third most common fracture among the elderly [1]. PHFs are typically high-energy fractures in younger male patients and fragility fractures in older female individuals [2]. These fractures can have significant implications for quality of life, particularly in younger patients, where they can often result in impaired shoulder function and chronic pain. For all these reasons PHFs present a significant clinical challenge, given their high incidence in elderly, fragile patients and the potential for postoperative complications [3]. The choice of anesthesia plays a pivotal role in optimizing surgical outcomes, minimizing opioid requirements, and enhancing patient recovery. The evolution of Regional Anesthesia (RA) techniques for upper limb surgeries has been driven by the need for improved postoperative pain control and reduced systemic complications associated with general anesthesia [4]. The first descriptions of Brachial Plexus (BP) anesthesia in the early 20th century relied on surface anatomical landmarks and blind needle placement [5]. The introduction of nerve stimulation techniques in the mid-20th century significantly improved the precision of regional blocks, allowing anesthesiologists to identify nerve structures based on muscle responses [5]. However, it was not until the late 20th and early 21st centuries that ultrasound guidance revolutionized the field. The use of real-time ultrasonography, first widely adopted in the 2000s, has dramatically increased the safety and efficacy of locoregional techniques by providing direct visualization of nerve structures, needle positioning, and local anesthetic spread [5]. Modern approaches to BP blocks, including interscalene, supraclavicular, infraclavicular, and axillary blocks, have been extensively studied for their analgesic benefits in PHFs surgeries [6]. Despite their effectiveness, potential complications such as phrenic nerve involvement and respiratory compromise in vulnerable patients have led to the exploration of alternative techniques [7].

Despite their central role in practice, robust high-quality evidence on RA specifically for PHF surgery remains scarce. Most available studies are small, heterogeneous, or extrapolated from other shoulder procedures. This gap underscores the need for a comprehensive synthesis of current knowledge, enriched by expert clinical perspectives, to guide real-world decision-making.

By combining available evidence with expert clinical perspectives, this review aims to address the current gap between limited scientific data and the growing clinical need for safe, effective, and patient-tailored RA strategies in PHF surgery.

## Materials and methods

### Literature search

We performed a structured non-systematic literature search to support this narrative review, following PRISMA 2020 for transparent reporting while acknowledging that no meta-analysis was planned. Searches were run in PubMed/MEDLINE, Embase, and Scopus covering January 1, 2000 to November 1, 2025. The following search terms were included: “proximal humerus”, “proximal humeral”, “PHF”, “fracture”, “regional anesthesia”, “regional anaesthesia”, “interscalene”, “supraclavicular”, “infraclavicular”, “costoclavicular”, “superior trunk”, “suprascapular”, “axillary nerve block”, “paravertebral”, “erector spinae”, and “ESPB”.

### Eligibility criteria

We included adult studies (≥ 18 years) reporting RA for surgical management of PHFs (e.g., ORIF, intramedullary nailing, hemiarthroplasty, reverse shoulder arthroplasty) with clinical outcomes. We excluded conference abstracts without full data, single-patient case reports (< 5 patients), anatomical/cadaveric or purely technical articles, non-surgical/ED-only analgesia, humeral shaft/distal fractures, and non-PHF shoulder surgery.

### Study selection

**Fig. 1:**
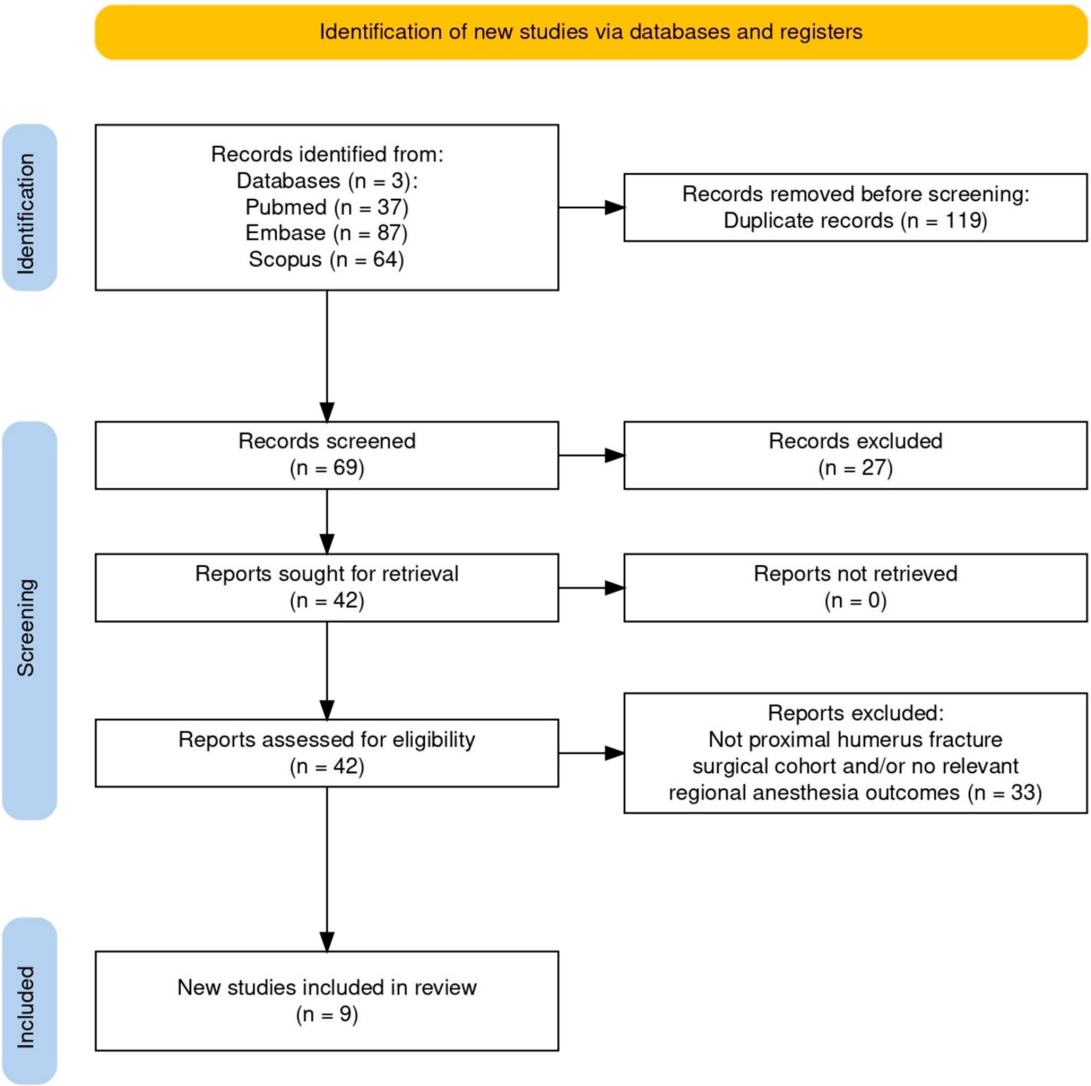
PRISMA flowchart. Note: additional references were cited for context and are not depicted in the PRISMA flow

After cross-database deduplication, 69 records underwent screening, of which 27 were excluded. We retrieved and assessed 42 full-text articles, excluding 33 with reasons (not PHF surgical cohort and/or no relevant RA outcomes). A total of 9 studies were included in the narrative synthesis. The PRISMA 2020 flow diagram (Fig. 1) details the process and exclusions.

### Characteristics of included studies

The studies meeting inclusion criteria were observational or clinical investigations focused on adult patients undergoing surgical repair of PHFs with RA. All included studies emphasized the scarcity of randomized controlled trials specifically addressing RA for PHF surgery, with most available data derived from small cohorts.

### Additional sources

In addition to database searching, we screened the reference lists of included studies and performed targeted citation tracking to ensure topical completeness. Sources not meeting our predefined eligibility criteria (e.g., narrative reviews, guidelines or consensus statements, anatomical or educational articles, single-patient case reports, editorials) were cited for background and context only. These were not included in the PRISMA flow diagram, which exclusively represents the identification and selection of eligible primary studies included in the narrative synthesis.

### Orthopedic overview

Given the clinical complexity of PHFs, we also considered it essential to include an orthopedic perspective, reviewing surgical fixation methods and the complications associated with each treatment modality. This preliminary overview was introduced to contextualize the anesthetic discussion, as the surgical approach directly influences block selection, analgesic strategies, and postoperative outcomes.

### Expert perspectives

The “Expert Perspectives” section (authored by G.S., G.R., D.P.S. and A.Coviello) reflects shared perspectives and practical insights gained through years of managing patients with PHFs in surgical and anesthetic settings. Although no formal consensus method (e.g., Delphi or RAND) was employed, the recommendations are based on multidisciplinary discussion and real-world practice.

## Summary of findings

At the end of the literature search, 9 studies met the inclusion criteria and were incorporated into the narrative synthesis. Most of these were characterized by small sample sizes and primarily addressed analgesic efficacy, technical feasibility, or respiratory safety, rather than long-term functional or rehabilitation-related outcomes. Only a minority of investigations specifically evaluated continuous regional anesthesia techniques or provided direct comparisons between diaphragm-sparing approaches and interscalene brachial plexus block in the context of PHF surgery. The main characteristics of the included studies are summarized in Table 1.

**Table 1:** Summary of the characteristics of the included studies

Author (Year)	Study Type	Population / Sample Size	Surgery Type	Regional Anesthesia Technique	Main Findings
Nah et al., [16]	Prospective pilot study	Small cohort	PHF fixation	Continuous interscalene block (home-based)	Reduced dynamic pain and good functional recovery; supports outpatient continuous block use
Sinha et al., [15]	Randomized controlled trial	62 patients	Proximal or mid-shaft humerus fixation	Superior trunk block vs. interscalene block	ST block provided similar analgesia to ISB with significantly lower incidence of hemidiaphragmatic paresis; no difference in opioid use or pain scores
Liu et al., [12]	Prospective double-blind study	30 patients (ASA I–II)	PHF surgery	Ultrasound-guided ISB with variable ropivacaine concentrations	Determined EC50 of 10 mL ropivacaine between 0.222–0.233%; defined optimal dosing for effective analgesia
Wang et al., [10]	Randomized controlled trial	80 elderly patients	PHF surgery (deltopectoral approach)	ISB + supraclavicular block ± T2 paravertebral block	Addition of T2 PVB improved anesthesia success and reduced GA conversion without added risk
Wang et al., [11]	Randomized controlled trial	Elderly patients	PHF fixation	ISB + thoracic paravertebral block	Study protocol describing combined ISB–PVB for improved block quality and reduced conversion to GA
Diwan et al., [14] (*Turk J Anaesthesiol Reanim*)	Case series	5 patients	PHF fixation	Continuous costoclavicular block with retrograde stimulating catheter	Effective postoperative analgesia and feasibility of diaphragm-sparing continuous technique
Lee et al., [23] (*J Clin Med*)	Narrative review	Adults with pulmonary compromise	Shoulder surgery	Superior trunk, suprascapular, axillary nerve blocks	Described RA strategies in patients with respiratory limitations; emphasized phrenic-sparing approaches
Iliaens et al., [9] (*Arch Orthop Trauma Surg*)	Systematic review	12 cohort studies (*n* = 248)	Shoulder and PHF surgery	Interscalene vs. supraclavicular, suprascapular, infraclavicular blocks	Limited evidence; highlighted need for diaphragm-sparing approaches
Egol et al., [8] (*Bull Hosp Jt Dis*)	Retrospective comparative study	92 patients	ORIF for PHF	ISB (± GA) vs. GA alone	RA improved pain control, enabled earlier mobilization, and yielded better functional outcomes

The included studies are briefly outlined below in chronological order, according to author, study design, and year of publication:

1. **Egol et al. (2014)** reported an observational cohort study assessing the effect of RA on perioperative outcomes in patients undergoing surgical repair of PHFs, describing improved early postoperative pain control and reduced opioid consumption compared with general anesthesia [8].
2. **Iliaens et al. (2019)** published a systematic review highlighting the limited quality and heterogeneity of the available evidence on RA for PHFs surgery. Despite these limitations, they concluded that RA provides effective postoperative analgesia, may reduce adverse events compared with general anesthesia, and could improve functional recovery, facilitate earlier mobilization and rehabilitation, and shorten recovery time. While ISBPB remained the most commonly used technique, they suggested potential advantages of continuous blocks and adjuvants, stressing the need to balance benefits against risks, particularly in elderly and frail patients. Overall, they emphasized that further well-designed randomized trials are required to confirm effects on functional outcomes, length of stay, and cost-effectiveness [9].
3. **Wang et al. (2020)** conducted a randomized controlled trial evaluating the addition of a T2 Paravertebral Block (PVB) to RA in elderly patients undergoing PHF surgery, reporting improved intraoperative anesthetic efficacy and postoperative pain control [10].
4. **Wang et al. (2022)** subsequently published the corresponding study protocol for the same randomized investigation (2020), further detailing the methodological rationale and design of the PVB approach in this patient population [11].
5. **Liu et al. (2022)** presented a dose-finding clinical study identifying the EC₅₀ of ropivacaine for ISBPB, indirectly supporting diaphragm-sparing strategies through reduced local anesthetic concentrations, although without direct assessment of clinical outcomes specific to PHF surgery [12].
6. **Lee et al. (2023)** authored a narrative review focusing on RA techniques for shoulder surgery in high-risk pulmonary patients, highlighting that diaphragm-sparing blocks can provide effective analgesia—and, in selected cases, surgical anesthesia—while minimizing phrenic nerve involvement. They emphasize individualized technique selection, careful limitation of local anesthetic volume, and balancing respiratory preservation with analgesic efficacy [13].
7. **Diwan et al. (2023)** described a small clinical case series investigating continuous costoclavicular block using a retrograde stimulating catheter technique for shoulder surgery, demonstrating technical feasibility, effective postoperative analgesia, and preservation of diaphragmatic function [14].
8. **Sinha et al. (2024)** reported the only randomized controlled trial directly comparing STB with ISBPB in patients undergoing humerus surgery, including PHFs, showing comparable analgesia with a significantly lower incidence of hemidiaphragmatic paresis in the STB group [15].
9. **Nah et al. (2025)** published a pilot feasibility study evaluating ambulatory catheter-based ISBPB for PHFs surgery, reporting improved short-term pain control and functional scores; however, inferences regarding outpatient management and home-based recovery were limited by the exploratory design and small sample size [16].

## Classifications of PHFs

The first classification of PHFs was published in 1934 by Ernest Codman. In 1970, Charles Neer published a system focusing on the displacement and number of fractured fragments (one/two/three/four-parts fractures) [17].

A different approach to the classification of PHFs is presented in Hertel’s binary fracture description model, which focuses on the fracture planes rather than the number of fragments. Hertel identified 12 basic fracture patterns, highlighting those that are likely to compromise vascular supply to the Humeral Head (HH). Key factors associated with an increased risk of ischemia and subsequent Avascular Necrosis (AVN) include metaphyseal extension of less than 8 mm and disruption of the medial hinge [18].

The Mayo-FJD classification described seven common fracture types: isolated fracture of the Greater or Lesser tuberosity (GT, LT), fractures involving the surgical neck without head deformity, fractures impacted in varus or in valgus, and fractures involving the HH where the head is dislocated (head dislocation), split (head-splitting), or depressed (head impaction). This system emphasizes fracture morphology, the degree of displacement, and shoulder joint stability, all critical factors in determining the appropriate treatment [19].

The AO/OTA classification system offered a more detailed classification, distinguishing between extra-articular and intra-articular fractures and further categorizing them based on fracture morphology and severity. While this system is valuable in research and complex cases, it can be cumbersome for routine clinical use [20].

## Conservative and surgical treatments

Multiple factors influence decision-making in the management of PHFs [21]. The surgeon must take into account the fracture pattern, the patient’s overall condition, and their own experience and skill level. Absolute indications for surgical treatment include fracture-dislocations, head-splitting fractures, pathological fractures, open fractures and neurovascular injuries [22].

In older patients, PHFs are often the result of low-energy trauma due to bone fragility. For patients over 85 years of age and/or those with low functional demands, surgery is rarely indicated [2, 3]. In younger patients, who typically experience PHFs due to high-energy trauma, surgery may be indicated even in less displaced fractures to allow early mobilization and improve short-term outcomes [2, 3]. When multiple surgical options are viable, the surgeon’s choice may be influenced by their familiarity with specific techniques [2, 3, 19].

### Conservative Treatment

Conservative treatment typically involves the use of a sling or shoulder immobilizer for 1–3 weeks, followed by early mobilization to prevent stiffness [23]. Pain control is crucial in the first week following a PHF, especially at night. Pain is often exacerbated when lying down, and many patients prefer to rest in a seated position. Early supervised physiotherapy, typically initiated within three weeks, is commonly recommended, although it does not appear to significantly impact final outcomes [24]. Fracture pattern, degree of displacement, and patient age significantly influence the outcomes of conservative treatment for PHFs [25]. Some studies have demonstrated favorable functional outcomes with non-operative management of displaced and multi-fragmentary fractures; however, it is widely acknowledged that these cases carry a higher risk of symptomatic malunion, non-union, and AVN [26–28]. In such instances, patients must be counselled about the possibility that a secondary “salvage” procedure may be required. These salvage surgeries are typically more challenging, carry worse functional outcomes, and are associated with increased complication rates [23].

### Surgical Treatment

Surgical management is considered for fractures with significant displacement, multiple fragments, or those involving the articular surface [29]. The main surgical strategies include Open Reduction and Internal Fixation (ORIF), Intramedullary Nailing (IMN), and prosthetic replacement [30]. The choice between these techniques is based on fracture type, patient factors such as age and bone quality, and the surgeon’s expertise [29].

**Fig. 2:**
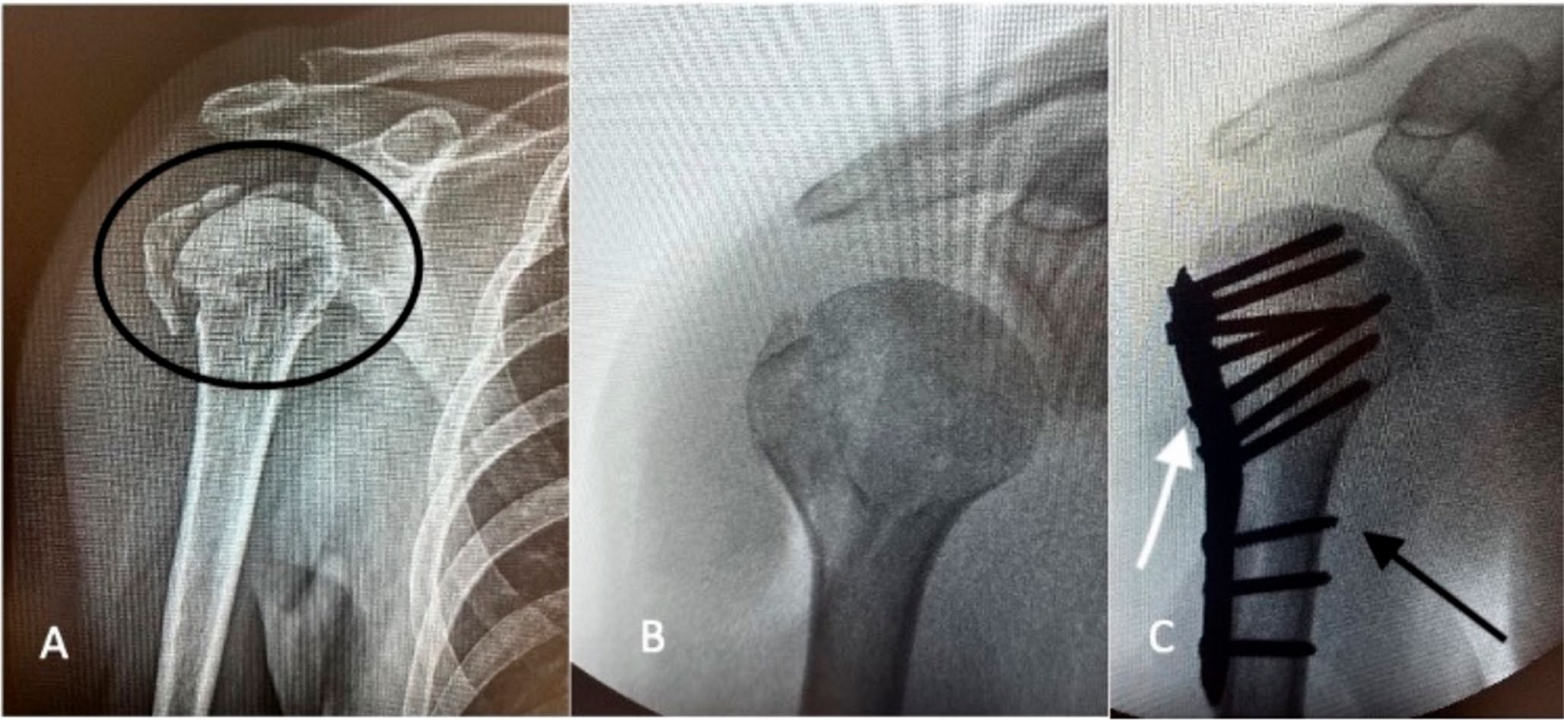
(**A**) Displaced four-part proximal humeral fracture (circled).(**B**) Intraoperative image showing fracture reduction. (**C**) Osteosynthesis with plate and screws; the white arrow indicates the fixation plate, and the black arrow indicates one of the screws

ORIF with locking plates is commonly used for displaced PHFs, especially in young or active patients [29, 31]. It offers angular stability, aiding fixation in osteoporotic bone. However, varus malalignment and missed medial hinge reduction raise AVN risk [29, 31]. Structural grafts may support reduction and healing (Fig. 2) [29–32].

**Fig. 3:**
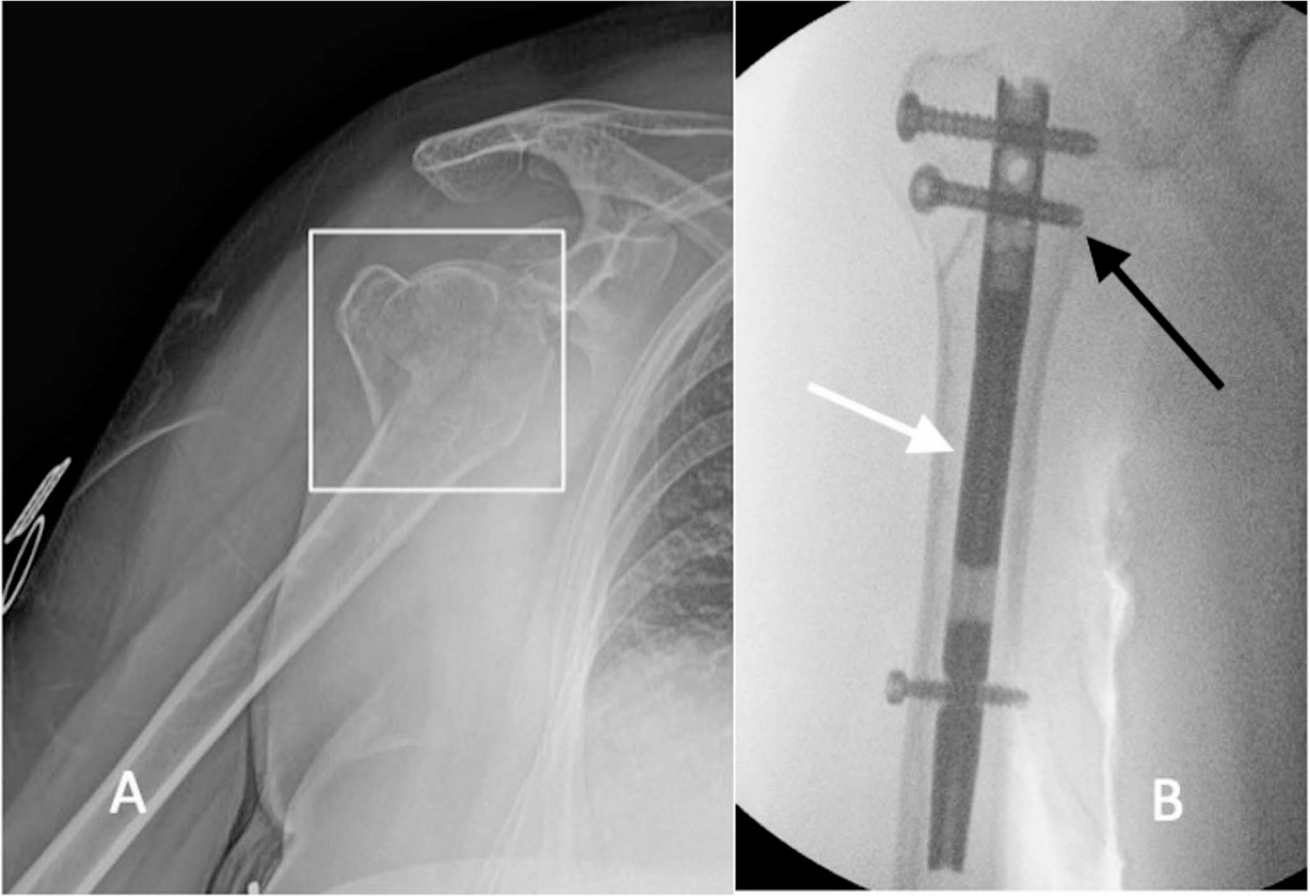
(**A**) Displaced proximal humeral fracture (highlighted within the box). (**B**) Osteosynthesis with an intramedullary nail; the white arrow indicates the nail, and the black arrow points to one of the locking screws

IMN offers a less invasive and biologically appealing alternative to plate fixation. It is particularly useful for two-part involving the surgical neck and selected three-parts PHFs. IMN provide load-sharing fixation, which can be advantageous in osteoporotic bone, and allow for earlier mobilization with less soft tissue disruption and better preservation of the periosteal blood supply compared to ORIF (Fig. 3) [29–32].

**Fig. 4:**
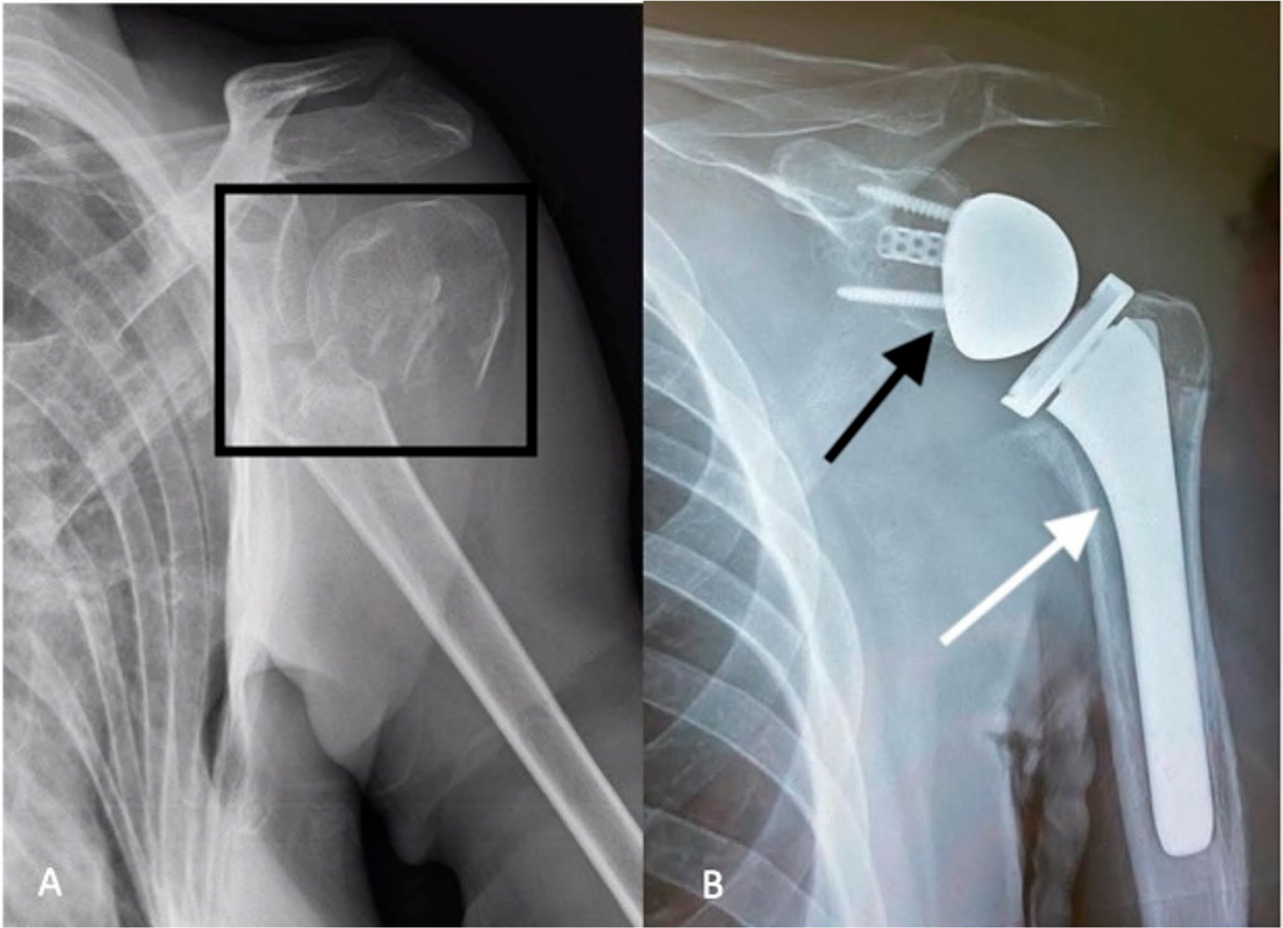
(**A**) Two-part proximal humeral fracture (highlighted within the box). (**B**) Prosthetic replacement; the black arrow indicates the glenosphere, and the white arrow points to the humeral stem

In cases of severe comminution, particularly in four-part fractures or “head split” fractures (with a high risk of AVN), prosthetic replacement is required. Arthroplasty is often used in complex fracture patterns in elderly patients, reducing the risk of reoperation in the event of osteosynthesis failure [29, 32]. Pain control after this procedure is essential to allow early mobilization: peripheral nerve blocks are a useful tool in the first days after surgery (Fig. 4) [33, 34].

## Treatment Complications

Complications associated with the management of PHFs depend on both the treatment approach and patient-related factors [3, 22]. An overview of implant-related and non-implant-related complications is provided in Table 2, while a summary is presented below to facilitate direct reference within the text.

**Table 2:** Complications based on treatment modality and correlation with the implant used

Treatment Modality	Non-Implant Related Complications	Implant Related Complications
Conservative	Malunion (13–23%) Non-union (1.1–10%) Avascular Necrosis (1–10%)	Not Applicable
Locking Plates	Avascular Necrosis (4.4%) Varus Malunion (6.8%) Non-union (1.5%)	Intra-articular Screw Penetration (9.5%) Subacromial Impingement (5%) Deep Infection (1.4%)
Intramedullary Nailing (IMN)	Malunion (9%) Avascular Necrosis (4%) Non-union (< 1%)	Loss of Reduction (10%) Subacromial Impingement (4%) Screw Cut-out (9%) Rotator Cuff Dysfunction (1.6%) Deep Infection (< 1%)
Hemiarthroplasty (HA)	Greater Tuberosity Complications (10%)	Prosthetic Migration (5%) Glenoid Erosion (56.8%) Deep Infection (2.5%)
Reverse Shoulder Arthroplasty (RSA)	Hematoma (2.6%) Nerve Injury (1.2%)	Scapular Notching (35%) Instability (4.7%) Glenoid Loosening (3.5%) Humeral Subsidence (1.3%) Deep Infection (3.8%) Fracture of the acromion/scapular spine (1.5%)

Implant-related complications primarily included screw loosening, plate displacement, and surgical site infection. These events occurred in a limited number of patients and were effectively managed through revision surgery or targeted antibiotic therapy, according to clinical indication [3, 22, 30].

Non-implant-related complications consisted mostly of transient nerve injury, postoperative pain requiring additional analgesic intervention, and minor pulmonary events such as atelectasis or mild pneumonia. These complications were self-limiting and resolved with appropriate conservative management [3, 22, 30].

Overall, the incidence and severity of complications were consistent with those described in previous reports, reinforcing the safety and reliability of both surgical fixation and RA techniques used in the management of PHFs [35].

### Comparison of surgical techniques for PHFs

PHFs, particularly in the elderly, pose a significant clinical challenge due to variations in fracture patterns, bone quality, and patient comorbidities [22]. While conservative treatment is appropriate for non-displaced fractures, surgical interventions such as ORIF, IMN, and prosthetic replacement (HA or RSA) are commonly indicated for more complex fractures, particularly in patients with higher functional demands [36]. Each of these surgical options carries specific complications risks, and management decisions should be tailored based on individual patient factors such as age, functional capacity, and the presence of comorbid conditions [22, 37]. A comparative evaluation of different strategies for PHFs is essential to guide clinical decision-making. These differences are summarized in Table 3.

**Table 3:** Comparison of surgical techniques for PHFs

Technique	Indications	Advantages	Limitations and Complications
ORIF (Locking Plates)	Young/active patients, good bone stock	Anatomic reduction, angular stability, familiarity	Risk of AVN, screw cut-out, need for precise technique
Intramedullary Nailing (IMN)	Two- or three-part fractures, osteoporotic bone	Minimally invasive, load-sharing, better vascular preservation	Malalignment, rotator cuff injury, reduced control of reduction
Hemiarthroplasty (HA)	Relatively younger patients with complex un-reconstructable PHF but no evidence of glenohumeral arthritis and preserved greater tuberosity and rotator cuff function	Consistent postoperative pain relief, shorter operative time, less blood loss and less technical complexity compared to RSA	Failed tuberosity healing had significantly worse functional outcomes, higher risk of revision surgery
Reverse Shoulder Arthroplasty (RSA)	Elderly, four-part fractures, poor bone quality	Pain relief, reduced reoperation, early mobility	Scapular notching, prosthetic complications, reduced rotation

## Shoulder joint and proximal humerus Innervation: regional distribution

**Fig. 5:**
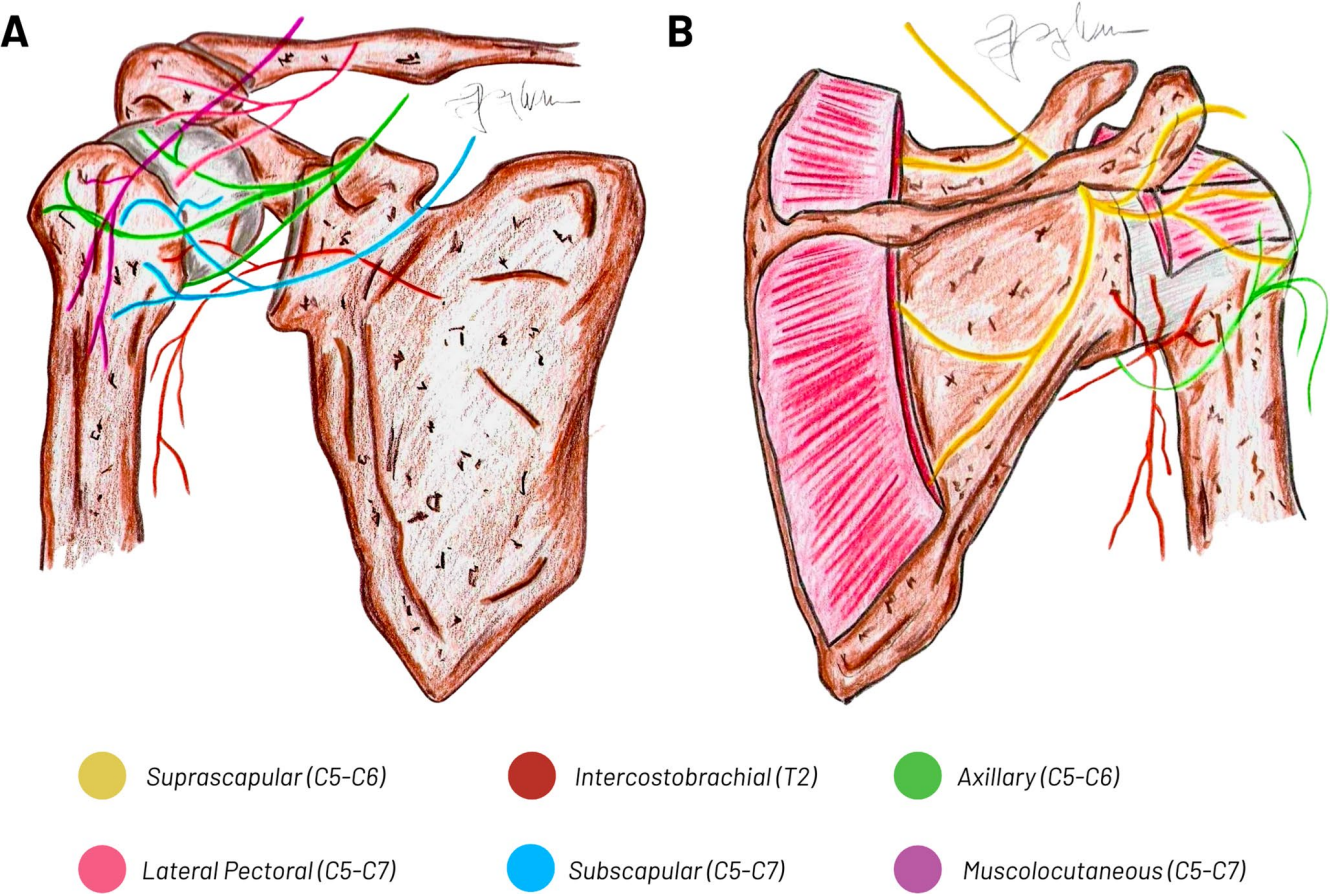
Schematic representation of the sensory innervation of the shoulder and proximal humerus. (**A**) Anterior aspect of the shoulder, illustrating the principal nerves contributing to anterior capsular and periosteal innervation: axillary nerve (green, C5–C6), intercostobrachial nerve (red, T2), lateral pectoral nerve (pink, C5–C7), subscapular nerves (blue, C5–C7), and musculocutaneous nerve (purple, C5–C7). (**B**) Posterior aspect of the shoulder, showing the main nerves involved in posterior joint and periarticular innervation: suprascapular nerve (yellow, C5–C6), axillary nerve (green, C5–C6), and intercostobrachial nerve (red, T2)

The sensory innervation of the shoulder joint and proximal humerus has a complex regional distribution, resulting from the overlapping contributions of multiple nerves originating from both the brachial and cervical plexuses [38]. Figure 5 provides a schematic overview of this innervation pattern, illustrating the principal peripheral nerves involved in the anterior and posterior capsular supply of the glenohumeral joint and proximal humerus.

- **Anterior Aspect**: The anterior shoulder and proximal humerus are mainly innervated by branches of the lateral pectoral nerve (C5-C7) and the musculocutaneous nerve (C5-C7), which provide sensory fibers to the anterior joint capsule and periosteum. The Axillary Nerve (AN) (C5-C6) also contributes to the anterolateral region, supplying sensation to the skin over the deltoid and parts of the anterior capsule.
- **Medial Aspect**: Sensory innervation to the medial shoulder and upper arm region primarily involves the branch of Intercostobrachial Nerve (ICBN) (T2), which crosses the axilla and provides cutaneous sensation to the medial upper arm and axillary region. This nerve does not contribute directly to the joint capsule but is relevant in shoulder pain and RA targeting the medial arm.
- **Lateral Aspect**: The lateral shoulder and proximal humerus receive significant sensory input from the AN (C5-C6), which innervates the skin overlying the deltoid muscle as well as the lateral joint capsule and periosteum. This nerve is crucial for lateral shoulder sensation and is a primary target for RA techniques like the interscalene and supraclavicular blocks.
- **Posterior Aspect**: The Suprascapular Nerve (SN) (C5-C6) is the main contributor to the posterior shoulder region, innervating the supraspinatus and infraspinatus muscles as well as the posterior joint capsule. Additionally, the upper and lower subscapular nerves (C5-C7) provide motor innervation to subscapularis and teres major muscles, with minor sensory contributions to the posterior capsule.
- **Superior Aspect**: The supraclavicular nerves from the Cervical Plexus (CP) (C3–C4) provide cutaneous sensory innervation to the skin over the clavicle, acromioclavicular joint, and the upper shoulder region. Though not directly involved in joint capsule innervation, these nerves influence superficial sensation and may affect RA approaches.

## RA techniques

### BP block

**Fig. 6:**
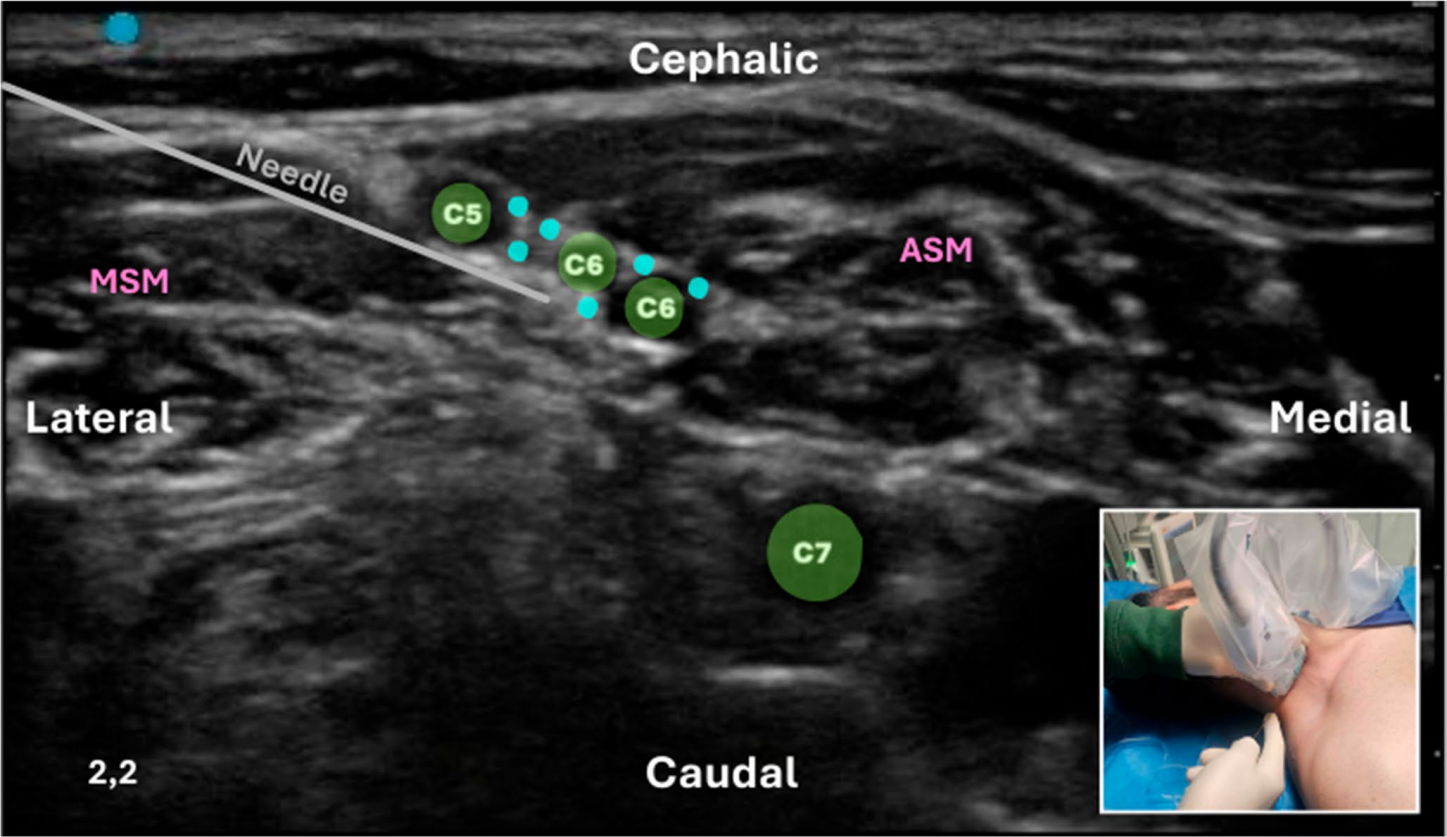
Ultrasound-guided conventional intrafascial ISBPB using a linear transducer with an in-plane lateral-to-medial needle approach (inset). The figure shows the interscalene space between the anterior scalene (ASM) and middle scalene (MSM) muscles, with the roots of the brachial plexus from C5 to C7. A conventional intrafascial technique is illustrated, in which the needle is advanced between the C5 and C6 roots, as indicated by the deposition of local anesthetic (light blue circles). This distribution occurs within a compartment that appears to be composed of multiple anatomical components, including loose connective tissue, thin fascial layers, and interfascial planes bounded by septa and adjacent structures

**Fig. 7:**
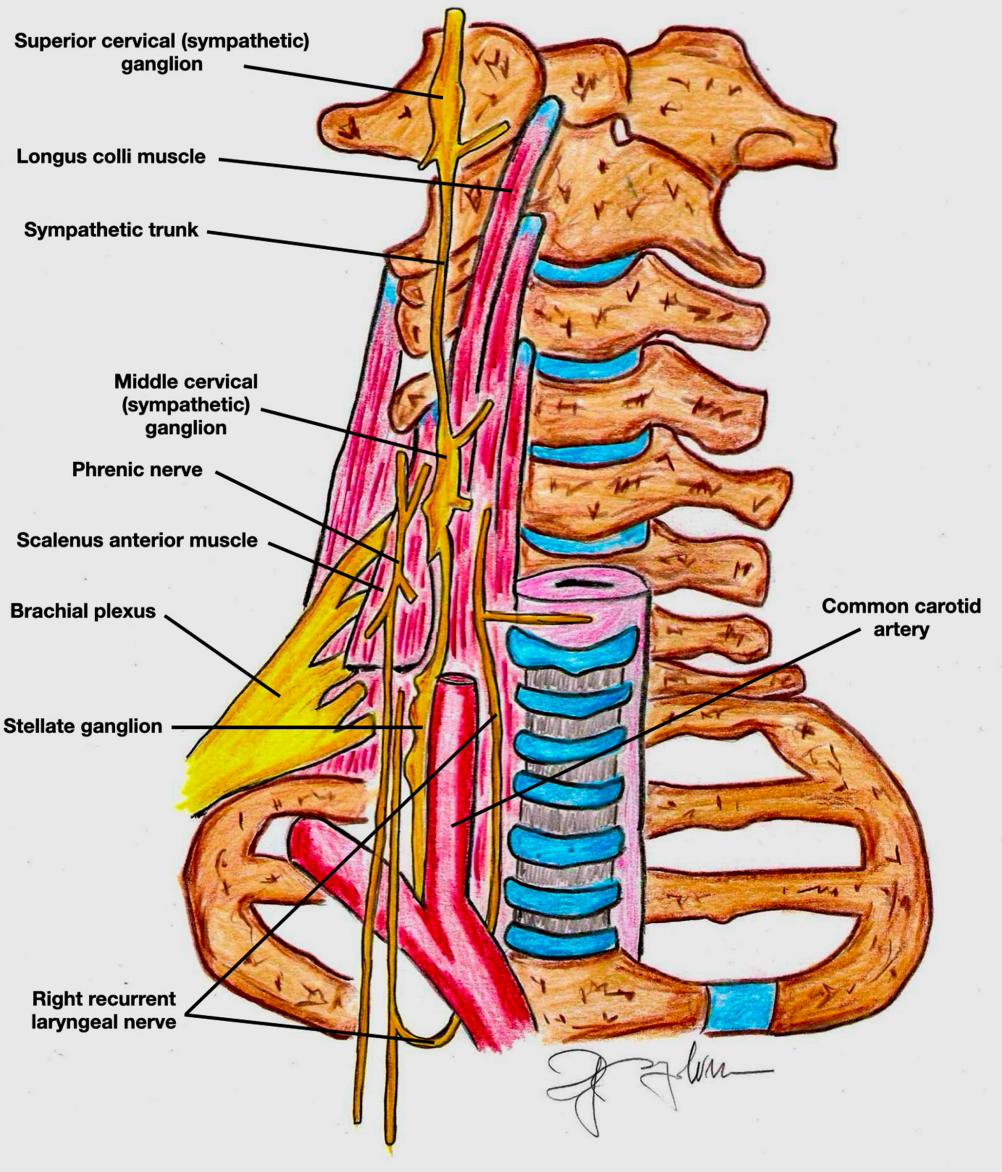
Schematic illustration of the cervical anatomy highlighting the close spatial relationship between the brachial plexus, the phrenic nerve, the recurrent laryngeal nerve, and the cervical sympathetic chain. This anatomical proximity explains the most common side effects associated with interscalene brachial plexus block and the potential pathways of local anesthetic spread. Phrenic nerve involvement may result in ipsilateral hemidiaphragmatic paralysis and respiratory impairment; recurrent laryngeal nerve block may cause dysphonia and airway symptoms; and spread to the sympathetic chain may lead to Horner’s syndrome, peripheral vasodilation, hypotension, and bradycardia

**Fig. 8:**
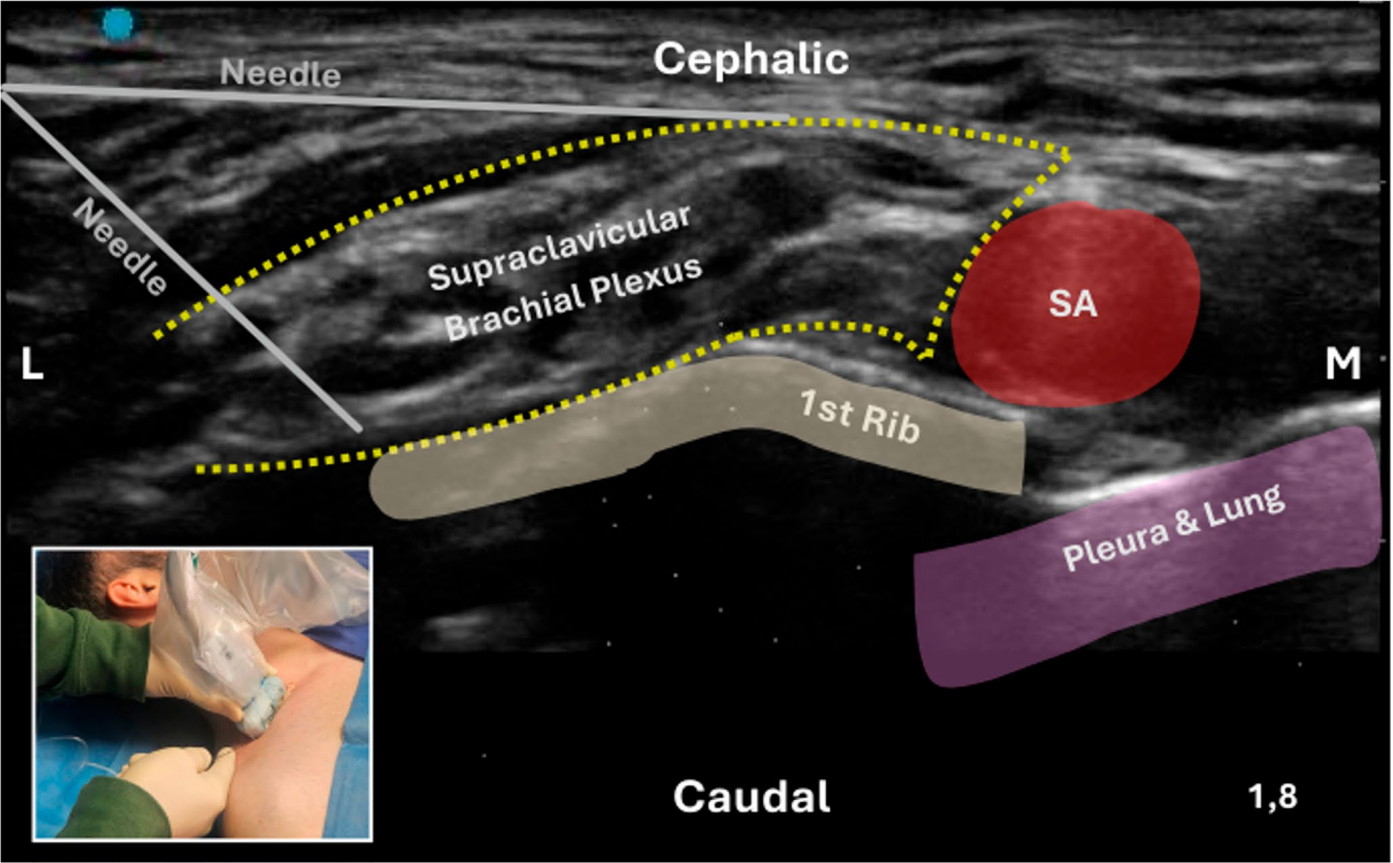
Ultrasound-guided supraclavicular brachial plexus block using a linear transducer with an in-plane lateral-to-medial needle approach (inset). The needle is advanced with one puncture and two minor redirections to ensure optimal spread of local anesthetic around the supraclavicular brachial plexus (yellow dashed line). Key sonoanatomical landmarks include the subclavian artery (SA), the first rib, and the pleura and lung, which should be carefully identified to minimize the risk of pneumothorax. Abbreviations: BP – Brachial Plexus; SA – Subclavian Artery; L – Lateral; M – Medial

**Fig. 9:**
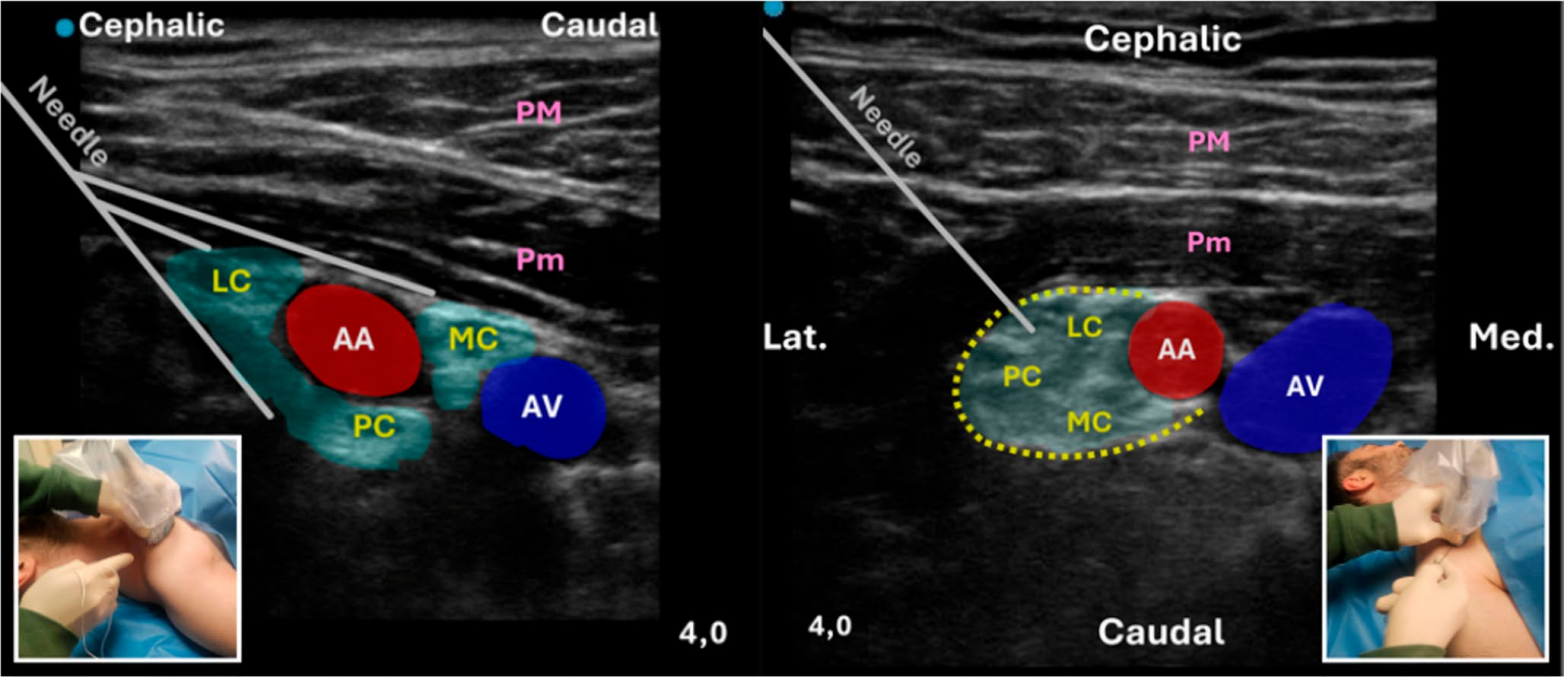
Ultrasound-guided infraclavicular brachial plexus block. On the left, the classic infraclavicular approach is shown: a high-frequency linear transducer is positioned transversely below the clavicle, parallel to the sternum, with the needle advanced in a cranial-to-caudal direction (in-plane). On the right, the costoclavicular approach is illustrated: the transducer is placed transversely below the midpoint of the clavicle to visualize the axillary artery and vein, with the three cords of the brachial plexus clustered laterally. The needle is advanced in-plane from lateral to medial toward the lateral aspect of the axillary artery. Insets show the corresponding patient and probe positions. Abbreviations: AA – Axillary Artery; AV – Axillary Vein; LC – Lateral Cord; MC – Medial Cord; PC – Posterior Cord; PM – Pectoralis Major; Pm – Pectoralis Minor; Lat. – Lateral; Med. – Medial

BP block remains the cornerstone of RA for upper limb surgeries. The most utilized approaches include:

- **Interscalene Brachial Plexus Block (ISBPB)**: The ISBPB was first described by Alon P. Winnie in the 1970s. Recognizing the anatomical advantages of the interscalene groove, Winnie developed a block that provided reliable anesthesia for the shoulder and upper arm. It is particularly advantageous for ORIF procedures; however, its major drawback is the high likelihood of phrenic nerve blockade, potentially leading to respiratory complications, especially in patients with preexisting pulmonary conditions [39]. Interscalene Space is a region bounded anteriorly by the Anterior Scalene (AS) muscle and posteriorly by the Middle Scalene (MS) muscle [40]. It represents a lateral continuation of the epidural space and is filled with loose fat and connective tissue, accommodating the ventral rami of spinal nerves from C5 to T1. Each ventral ramus is surrounded by an epineural sheath, which may contain varying amounts of fatty tissue. The tissue around the ventral rami is filled with fat and loose connective tissue without a specific sheath (Fig. 6). The prevertebral fascia covers both the local nerve structures and the prevertebral muscles (longus colli and scalene muscles), extending to the lateral margin of the interscalene space and the phrenic nerve. Injecting large volumes of local anesthetic for ISBPB may spread to the phrenic nerve and medially into an area filled with loose connective tissue, potentially leading to blockade of the recurrent laryngeal nerve, the sympathetic chain, or components of the autonomic cardiac innervation (Fig. 7) [40].

- **Supraclavicular Brachial Plexus Block**: The supraclavicular block was first described by Diedrich Kulenkampff in 1911 [41]. He recognized that injection of local anesthetic above the clavicle, in close proximity to the BP, could provide dense anesthesia of most of the upper limb [39]. The supraclavicular approach offers extensive coverage of the shoulder and arm, with a lower incidence of respiratory impairment compared with the interscalene block. However, complete anesthesia of the entire upper limb cannot be achieved with any BP block, as the ICBN—originating from the T2 intercostal nerve—lies outside the BP and is therefore consistently spared [6]. In the supraclavicular region, the ventral rami of the BP emerge to form nerve trunks: the C5/C6 branches combine into the Superior Trunk (ST), C7 into the middle trunk, and C8/T1 into the inferior trunk [42]. The posterior triangle of the neck is bounded anteriorly by the posterior border of the sternocleidomastoid muscle, posteriorly by the anterior border of the trapezius muscle, and inferiorly by the clavicle. The nerve structures are positioned mediocaudally and remain covered by the prevertebral fascia, which fuses with the fascia of the subclavius muscle at the clavicle level. The long thoracic and dorsal scapular nerves arise directly from the ventral rami of C5-C7 (with C8 involved in 8% of cases), pass through the MS muscle, and innervate the muscles of the laterodorsal thoracic wall. The nerve trunks are particularly relevant for their topographical relationship with the subclavian artery (Fig. 8). The superior and middle trunks run cranially to the artery, transitioning laterally at the caudal level, while the inferior trunk is positioned dorsally near the so-called “corner pocket.” Connective tissue layers, possibly in continuity with the Zuckerkandl-Sebileau ligaments, may separate the inferior trunk from the superior and middle trunks. These ligamentous structures may act as anatomical barriers, potentially limiting the spread of local anesthetic and thereby influencing the extent and consistency of analgesia [43]. At the clavicular level, two additional branches emerge from the BP: the SN and the subclavian nerve. The SN, the most lateral nerve in this region, is clearly visible on ultrasound as it courses laterally parallel to the clavicle. It passes through the scapular notch beneath the superior transverse ligament and innervates the supraspinatus and infraspinatus muscles, as well as providing sensory fibers to the acromioclavicular and glenohumeral joints [43].

- **Infraclavicular Brachial Plexus Block**: This alternative technique targets the cords of the BP, offering consistent anesthesia with fewer respiratory side effects. It is particularly useful for patients in whom avoidance of diaphragmatic paralysis is critical [42]. Infraclavicular Fossa, also known as Mohrenheim’s fossa, is a region bounded by the clavicle, the deltoid muscle, the pectoralis major muscle, the upper part of the thorax, and the midclavicular line. The clavipectoral fascia, laterally continuous with the prevertebral fascia and connected to the fascia of the subclavius muscle, divides this area into a superficial and deep layer. The cephalic vein, which drains into the subclavian vein, is the most lateral structure of the deep layer of the infraclavicular fossa. The thoracoacromial artery, originating from the subclavian artery, is also visible in this region. Additionally, the lateral and, in rare cases, medial pectoral nerves are located superficially, innervating the pectoralis major and minor muscles. The lateral cord of the BP is the most superficial nerve structure in the deep layer of the infraclavicular fossa, located anterolaterally relative to the subclavian artery. The posterior cord is positioned dorsolaterally, while the medial cord is located dorsally to the artery. Behind the pectoralis minor, this arrangement changes with a pronation-style winding of all three cords around the artery. Due to the high prevalence of anatomical variations, ultrasound imaging may occasionally display more than three nerve structures due to the early division of cords or trunks. The infraclavicular block has the advantage of reducing complications when performed with ultrasound and is particularly well-suited for catheter placement. However, a key drawback is that the BP is located deeper, and the steeper angle of approach can make it challenging to visualize the anatomy and manipulate the needle simultaneously, unless the healthcare provider is highly experienced in the procedure. For the same reasons, this technique can also be more difficult in obese patients [44, 45]. Several ultrasound-guided approaches have been described to the infraclavicular block, each offering specific advantages in terms of visualization and anesthetic spread:
- **Classic Approach**: The ultrasound probe is positioned transversely, parallel to the sternum, with the marker oriented cranially above the clavicle, and the needle is advanced in a cranial-to-caudal direction. This method allows good visualization of the BP and surrounding vessels but can be challenging in obese patients (Fig. 9) [45].

- **RAPTIR Approach (Retroclavicular Approach to the Infraclavicular Region)**: The needle is introduced posterior to the clavicle in an anteromedial direction. This technique provides a more linear trajectory, facilitating needle handling and catheter placement for continuous analgesia [46].
- **Costoclavicular Approach**: With the patient supine and the head turned contralaterally, a high-frequency linear probe was placed transversely below the midpoint of the clavicle to visualize the axillary artery and the three cords of BP within the costoclavicular space. Using an in-plane lateral-to-medial approach, the needle was advanced toward the lateral aspect of the artery. This technique provides reliable anesthesia for surgeries below the mid-humerus, with rapid onset, consistent spread, and a lower risk of pneumothorax compared with the classical infraclavicular approach (Fig. 9) [45].
- **Parasagittal Approach**: The ultrasound probe is positioned parallel to the sternum, allowing a vertical visualization of the BP. This approach can enhance the spread of the anesthetic around the nerves [45].
- **Coracoid Approach**: The ultrasound probe is positioned near the coracoid process, with the needle advanced in a lateral-to-medial direction toward the BP. This technique allows for a more targeted anesthetic infiltration [45].
- **Continuous Peripheral Nerve Catheters**: The insertion of continuous nerve catheters at the BP allows for prolonged infusion of local anesthetics, significantly improving pain control over several days [43–45].

BP Block techniques are summarized in Table 4.

**Table 4:** Summary of a BP Block techniques

Technique	Coverage	Advantages	Disadvantages
ISBPB	Shoulder, upper arm	Effective for shoulder surgery (e.g., ORIF), high success rate	High risk of phrenic nerve blockade, potential respiratory and hemodynamic complications
Supraclavicular Block	Upper limb (ICBN-sparing)	Dense anesthesia of most of the upper limb	Risk of pneumothorax, risk of accidental puncture of subclavian vessels
Infraclavicular Block	Lower brachial plexus, forearm, hand	Fewer respiratory side effects, good for catheter placement.	More technically challenging, may not provide complete anesthetic coverage for humeral surgery.
Continuous Peripheral Nerve Catheters	Variable depending on site	Prolonged pain control, opioid-sparing	Requires catheter maintenance

#### ISBPB: anatomical variability

**Fig. 10:**
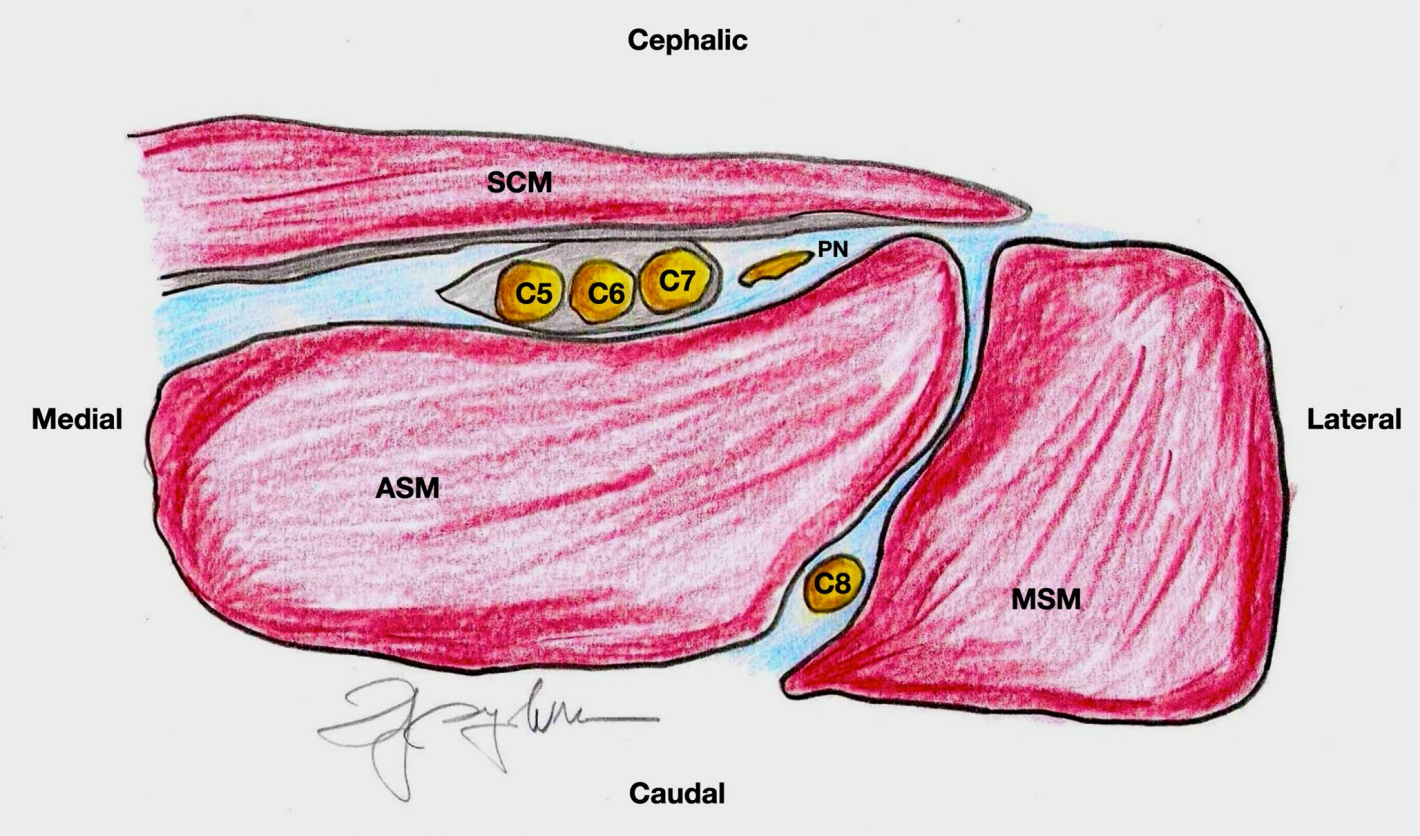
Cross-sectional view of an anatomical variant of the interscalene brachial plexus. In this configuration, the brachial plexus lies entirely on the surface of the anterior scalene muscle (ASM), rather than in the interscalene space between the anterior and middle scalene muscles (MSM). The phrenic nerve (PN) courses over the anterior surface of the ASM. This anatomical arrangement is associated with an increased likelihood of phrenic nerve involvement during interscalene brachial plexus block. Abbreviations: ASM – Anterior Scalene Muscle; MSM – Middle Scalene Muscle; SCM – Sternocleidomastoid Muscle; C5–C8 – Cervical Nerve Roots 5 to 8; PN - Phrenic Nerve.

Through the ultrasound-guided ISBPB, the commonly described anatomical relationship of the BP lying between the AS and MS muscles was found in only 60% of instances. On this basis, the anatomical variations in BP anatomy with respect to scalene muscles are common. Scalenus minimus was present in 46% of instances (bilateral in 14 cadavers). The most common variation was the penetration of the AS by the C5 and/or C6 ventral rami. The C5 and C6 roots may fuse before piercing AS (15% cases, bilateral in 4 cadavers), or the C5 root alone pierces the belly of AS (13% cases, bilateral in 3 cadavers). The roots also may pierce AS independently (6% cases, bilateral in 1 cadaver). In 3%, the C5 root was found to be completely anterior to AS [47]. Moreover, based on our individual ultrasound scanning experiences and expertise, we have found the BP entirely lying horizontally on the ventral surface of AS where the phrenic nerve runs (Fig. 10). These variations may pose several concerns not only for nerve stimulation-based approaches to ISBPB, but also for the concrete possibility to involve the phrenic nerve even if approached with the ultrasound-based technique due to the spread of local anesthetic whether the block is performed outside or inside the fascia involving the plexus. In the horizontal position of the plexus, the root of C5 is placed in front of the advancement of the needle tip making it difficult to pierce the fascia and increasing the possibility of anesthetic spread over the phrenic nerve. Conversely, in the case of blockage beyond the fascia, the needle tip will have to pierce the fascia while paying attention to the root of C5 and subsequent diffusion of local anesthetic will be unpredictable with increased chance of wetting the phrenic nerve [48–50].

In some cases a simultaneous diaphragmatic and BP stimulation followed by a supraclavicular approach has occurred due to the presence of the phrenic nerve inside the supraclavicular BP [51].

Last but not least, anatomical variability of the phrenic nerve and BP are encountered more frequently than expected. This results in the increased possibility of pharmacological paralysis of the nerve due to the unpredictability of its anatomical pathway [52, 53].

### Diaphragm-sparing techniques

**Fig. 11:**
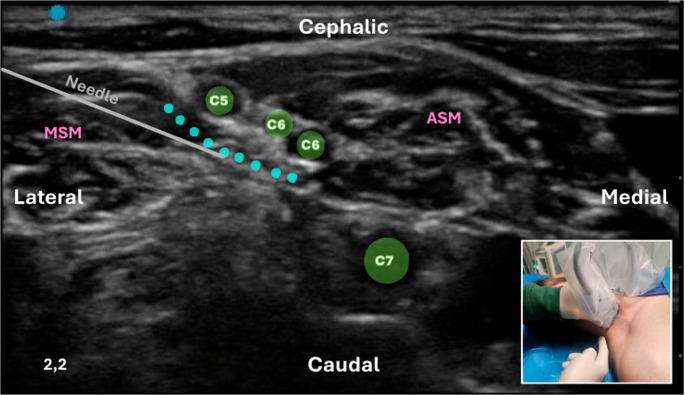
Ultrasound-guided extrafascial ISBPB using a linear transducer with an in-plane lateral-to-medial needle approach (inset). The figure shows the interscalene space between the anterior scalene (ASM) and middle scalene (MSM) muscles, with the brachial plexus roots from C5 to C7. In contrast to the conventional intrafascial approach, an extrafascial technique is illustrated, in which the needle is not advanced between the C5 and C6 roots but is positioned more laterally, adjacent to the MSM, outside the compartment containing them, as indicated by the deposition of local anesthetic (light blue circles). Abbreviations: ASM – Anterior Scalene Muscle; MSM – Middle Scalene Muscle; C5–C7 – Cervical Nerve Roots 5 to 7

**Fig. 12:**
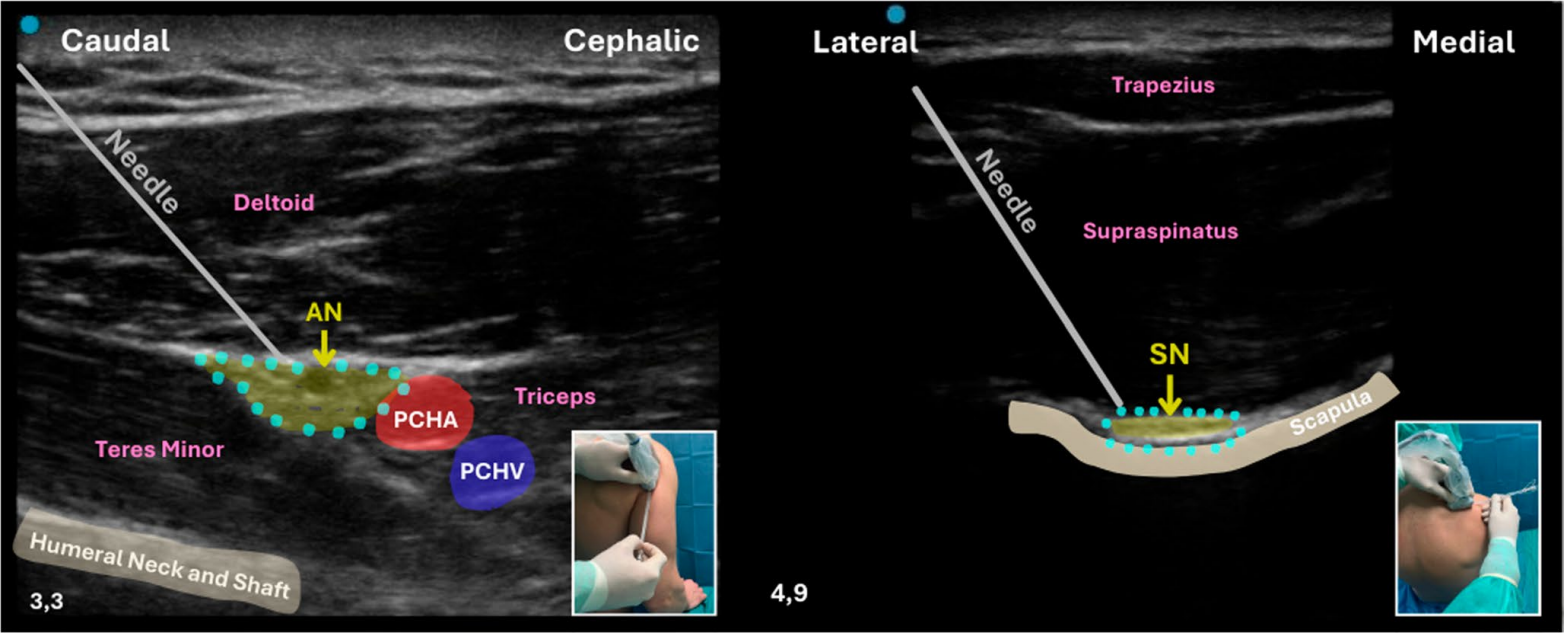
Ultrasound-guided combined AxNB + SSNB. On the left, axillary nerve block performed via a posterior approach, with an in-plane needle trajectory directed from caudal to cranial. The axillary nerve (AN) is visualized near the posterior circumflex humeral artery (PCHA) and vein (PCHV), between the deltoid and teres minor muscles. On the right, suprascapular nerve block performed through a lateral-to-medial in-plane needle approach at the supraspinous fossa, targeting the suprascapular nerve (SN) beneath the supraspinatus muscle. In both panels, the blue dots indicate the target area for local anesthetic injection. Insets show the corresponding transducer and needle positioning. Abbreviations: AN – Axillary Nerve; SN – Suprascapular Nerve; PCHA – Posterior Circumflex Humeral Artery; PCHV – Posterior Circumflex Humeral Vein

In addition to BP blocks, other techniques can be utilized to enhance analgesia while preserving the function of the hemidiaphragm:

- **Extrafascial injection for ISBPB**: The concept of the prevertebral fascia as a true “BP sheath” is not supported by macroscopic anatomical studies nor described in standard anatomical textbooks [54]. Nevertheless, the term *sheath* has been widely adopted in RA literature and clinical practice, primarily as a descriptive and operational construct, leading to the distinction between so-called “intrafascial” and “extrafascial” needle placement during ISBPB [55]. In clinical practice, extrafascial needle placement is commonly interpreted as positioning the needle tip adjacent to the MS muscle, with the intent of depositing local anesthetic outside the presumed fascial envelope surrounding the BP. The underlying rationale is to limit medial and ventral spread of the injectate, thereby reducing the risk of phrenic nerve involvement, hemidiaphragmatic paresis, and other block-related complications [56]. However, this rationale implicitly assumes the presence of a consistent and continuous fascial barrier, an assumption that is not supported by current anatomical evidence [54, 55]. Anatomical and imaging studies suggest that such a discrete BP sheath is inconsistently present or not reliably identifiable, and that local anesthetic spread is largely governed by variable connective tissue planes and individual anatomical differences, rather than by a well-defined fascial compartment [57]. As a consequence, when the needle is positioned extrafascially—particularly when aligned with the MS muscle—the injection may effectively be intramuscular, with the muscle’s own thin fascia acting as a partial barrier (Fig. 11). This may result in slower or less predictable spread of local anesthetic toward the plexus, potentially requiring higher volumes or longer onset times to achieve adequate neural blockade, and without guaranteeing avoidance of phrenic nerve involvement [58]. In this context, Sharapi et al. (2024) performed a systematic review and meta-analysis comparing extrafascial versus intrafascial injection techniques for ISBPB. The authors reported comparable block success rates and analgesic efficacy between the two approaches, while observing a significantly lower incidence of hemidiaphragmatic paresis and respiratory complications with the extrafascial technique. Conversely, intrafascial injection, performed within the perineural compartment near the C5–C6 roots, was associated with a significantly faster onset of both sensory and motor block compared with the extrafascial approach (median onset ≈ 4 vs. 6 min for shoulder abduction loss), likely reflecting more direct and homogeneous local anesthetic spread around neural elements. By contrast, extrafascial injection, typically performed lateral to the plexus and adjacent to the MS muscle, results in a slower block onset, as anesthetic diffusion toward the plexus may be partially constrained by fascial planes and the MS muscular fascia. Overall, these findings suggest a trade-off between faster onset with intrafascial techniques and improved respiratory safety with extrafascial injection, supporting a patient-tailored approach, particularly in individuals at increased risk of clinically relevant respiratory compromise [59].

- **Combined Axillary and Suprascapular Nerve Blocks**: A combined Axillary and Suprascapular Nerve Block (AxNB + SSNB) has emerged as a promising technique for shoulder surgeries, including PHFs repair (Fig. 12) [60–62].

The SN (C5-C6) arises from the ST and provides sensory innervation to approximately 70% of the shoulder joint, including the glenohumeral joint, acromioclavicular joint, and posterior shoulder capsule. The SSNB is an effective regional technique for managing shoulder pain, with two main approaches: posterior and anterior. Both provide analgesia by targeting the nerve that innervates much of the shoulder joint. The posterior approach is more traditional and widely used, while the anterior approach, performed near the ST, is gaining popularity for its potential advantages. The choice between them depends on factors such as the specific clinical situation, patient anatomy, and practitioner expertise. However, evidence is still evolving, and choice of technique should consider both anatomical factors and clinical priorities [60–62]. The AN (C5-C6) emerges from the posterior cord of the BP, supplying the deltoid muscle, teres minor, and lateral aspect of the shoulder. By combining these two blocks, anesthesia covers both the anterior and posterior aspects of the shoulder, ensuring effective pain control with minimal respiratory impairment [60, 62]. Sun et al. (2021), in a meta-analysis of randomized controlled trials, compared the combined AxNB + SSNB with the ISBPB for arthroscopic shoulder surgery. The authors found that the combined approach provided comparable postoperative analgesia and patient satisfaction, while significantly reducing the incidence of hemidiaphragmatic paralysis and other respiratory complications. These results support AxNB + SSNB as an effective and safer alternative, particularly for patients at risk of respiratory compromise or contraindicated for ISBPB [63].

**Fig. 13:**
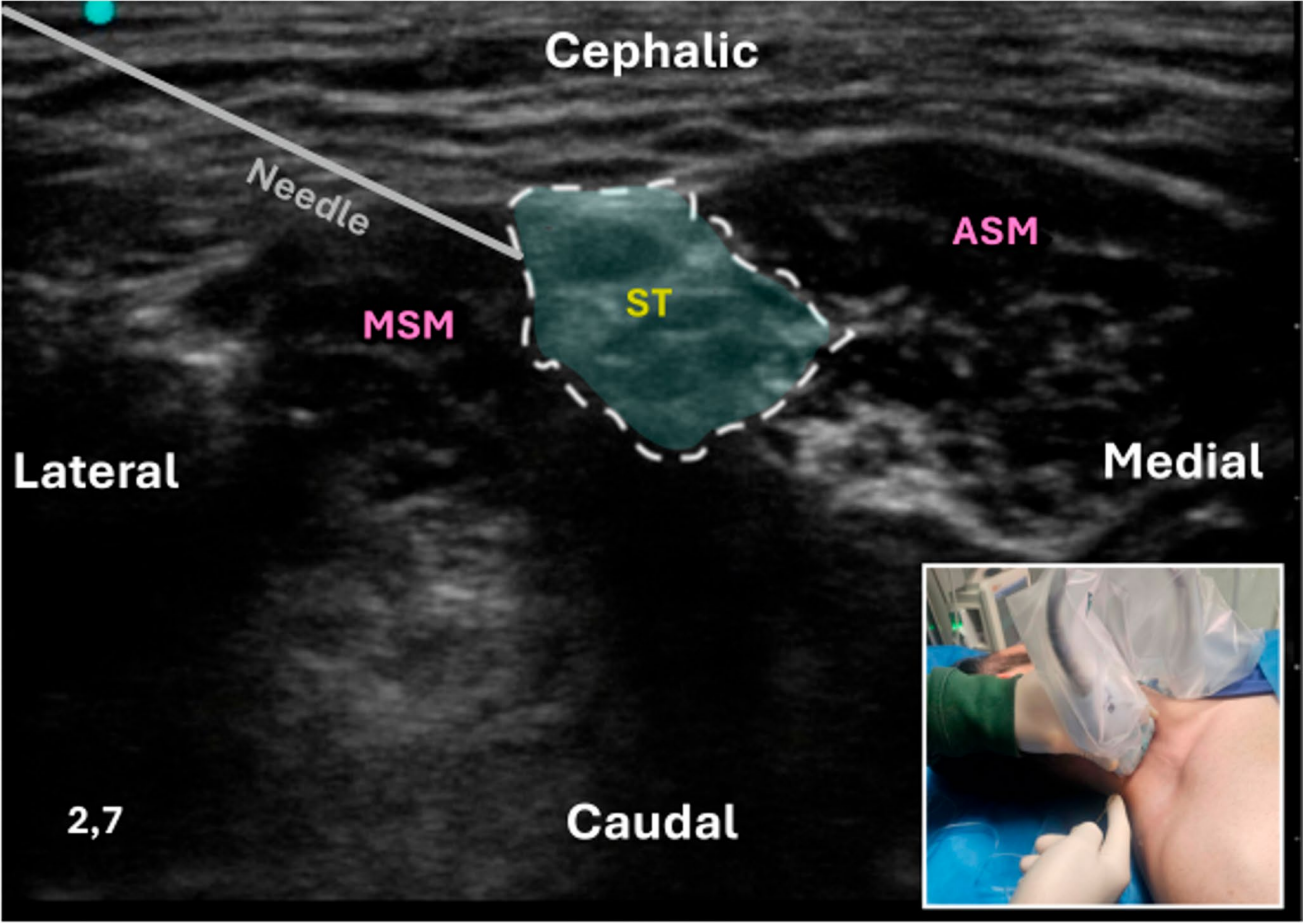
Ultrasound-guided STB. The block is performed at a lower level than the interscalene block, identifying the superior trunk (outlined by the white dashed lines), formed by fusion of C5 and C6 nerve roots and located between the anterior scalene (ASM) and middle scalene (MSM) muscles. The needle is advanced in-plane from lateral to medial. The blue-shaded area within the dashed lines represents the spread of local anesthetic around the superior trunk. The inset shows the corresponding transducer and needle position

**Fig. 14:**
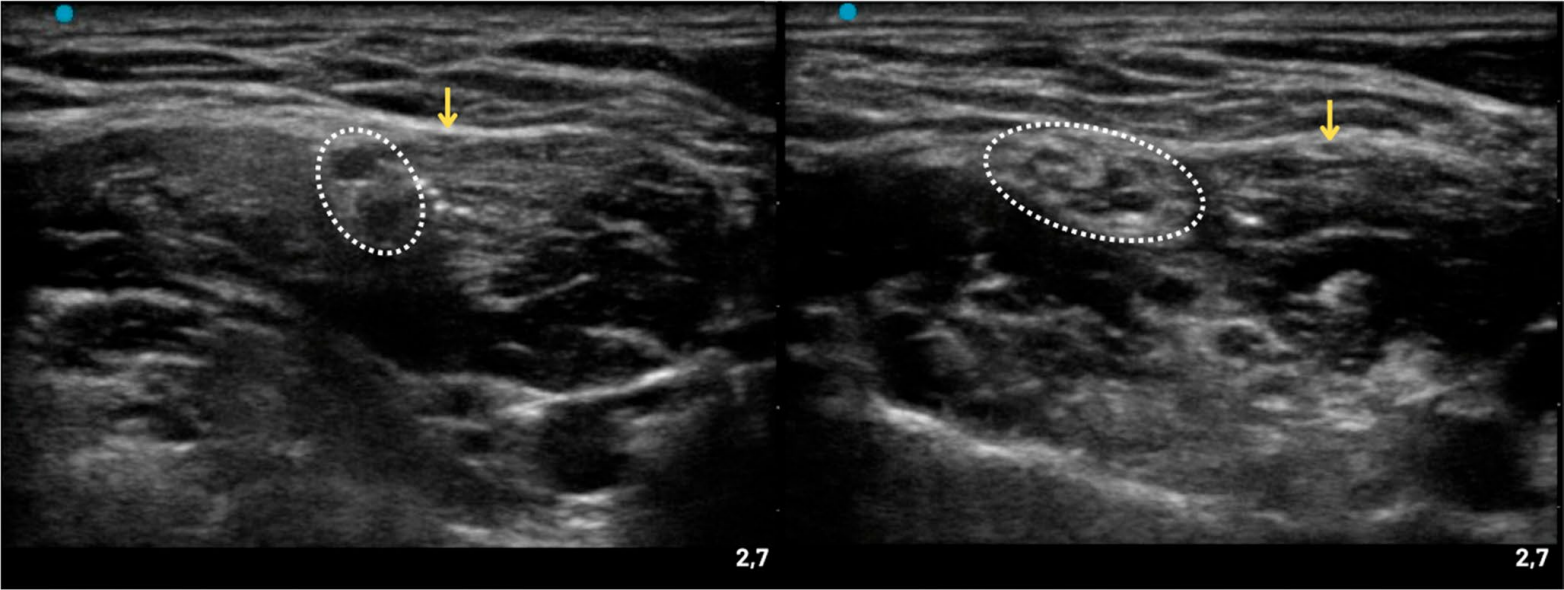
Ultrasound images comparing interscalene brachial plexus (left) and superior trunk (right). Neural structures are highlighted by white dotted circles. The yellow arrow indicates the relationship with the phrenic nerve. In the interscalene view, the plexus lies in close proximity to the phrenic nerve, whereas the superior trunk is located at a greater distance, providing a larger safety margin and a lower likelihood of phrenic nerve involvement

- **STB**: The local anaesthetic is deposited around the ST (formed by fusion of C5 and C6 nerve roots) (Fig. 13).

- Compared with traditional ISBPB, STB has been consistently associated with a significantly lower incidence of complete or partial hemidiaphragmatic paresis, while providing noninferior postoperative analgesia in shoulder and upper limb surgery [64]. Figure 14 illustrates the different sonoanatomical relationships between the interscalene BP and the ST with the phrenic nerve, highlighting the greater distance between the ST and the phrenic nerve that underlies the improved respiratory safety of STB. Randomized clinical trials have demonstrated that STB achieves analgesic efficacy comparable to ISBPB, with similar pain scores at 24 h and comparable opioid consumption, supporting its effectiveness for both surgical anesthesia and postoperative pain control [65–67]. A key advantage of STB is the marked reduction in hemidiaphragmatic paralysis, with reported rates decreasing from approximately 70–100% with ISBPB to 5–16% with STB, resulting in improved preservation of pulmonary function. In addition, STB has been associated with a lower incidence of block-related adverse effects, including hoarseness and Horner’s syndrome, and with higher patient satisfaction and fewer respiratory complaints [66]. Regarding block performance, most studies report noninferior quality of surgical anesthesia with STB, although isolated trials have described a slightly higher rate of incomplete block or need for supplementation compared with ISBPB, a finding not consistently reproduced across studies [68]. Onset time and motor block characteristics are generally similar between techniques, although a marginally faster onset has been reported with ISBPB in some series [68]. Overall, STB represents a diaphragm-sparing alternative to ISBPB, offering comparable analgesia with a substantially improved respiratory safety profile, and may therefore be preferable in patients at increased risk of pulmonary complications.

**WALANT (Wide Awake Local Anesthesia No Tourniquet)**: WALANT employs a combination of local anesthetic (lidocaine) and epinephrine to achieve surgical anesthesia and hemostasis without the use of a tourniquet or general anesthesia. This technique allows patients to remain fully awake, thereby avoiding airway manipulation, systemic anesthetic exposure, and tourniquet-related discomfort. In the setting of PHF surgery, WALANT may reduce perioperative cardiopulmonary risk, minimize intraoperative bleeding, and improve visualization of anatomical structures, facilitating fracture reduction and fixation. Additionally, real-time patient cooperation enables immediate assessment of shoulder motion and fixation stability during the procedure. Evidence supporting WALANT for PHF surgery is currently limited to isolated clinical reports; however, the case described by Shepard et al. (2024) demonstrated the feasibility and safety of this approach in carefully selected patients, suggesting a potential role for WALANT in frail individuals or those with significant contraindications to general anesthesia [69].

### Fascial plane blocks

Posterior thoracic wall blocks, including the Erector Spinae Plane Block (ESPB) and the PVB, have gained attention since they could provide longer-lasting analgesia to the shoulder and upper limb while minimizing the risks associated with traditional ISBPB, such as phrenic nerve paralysis.

**Fig. 15:**
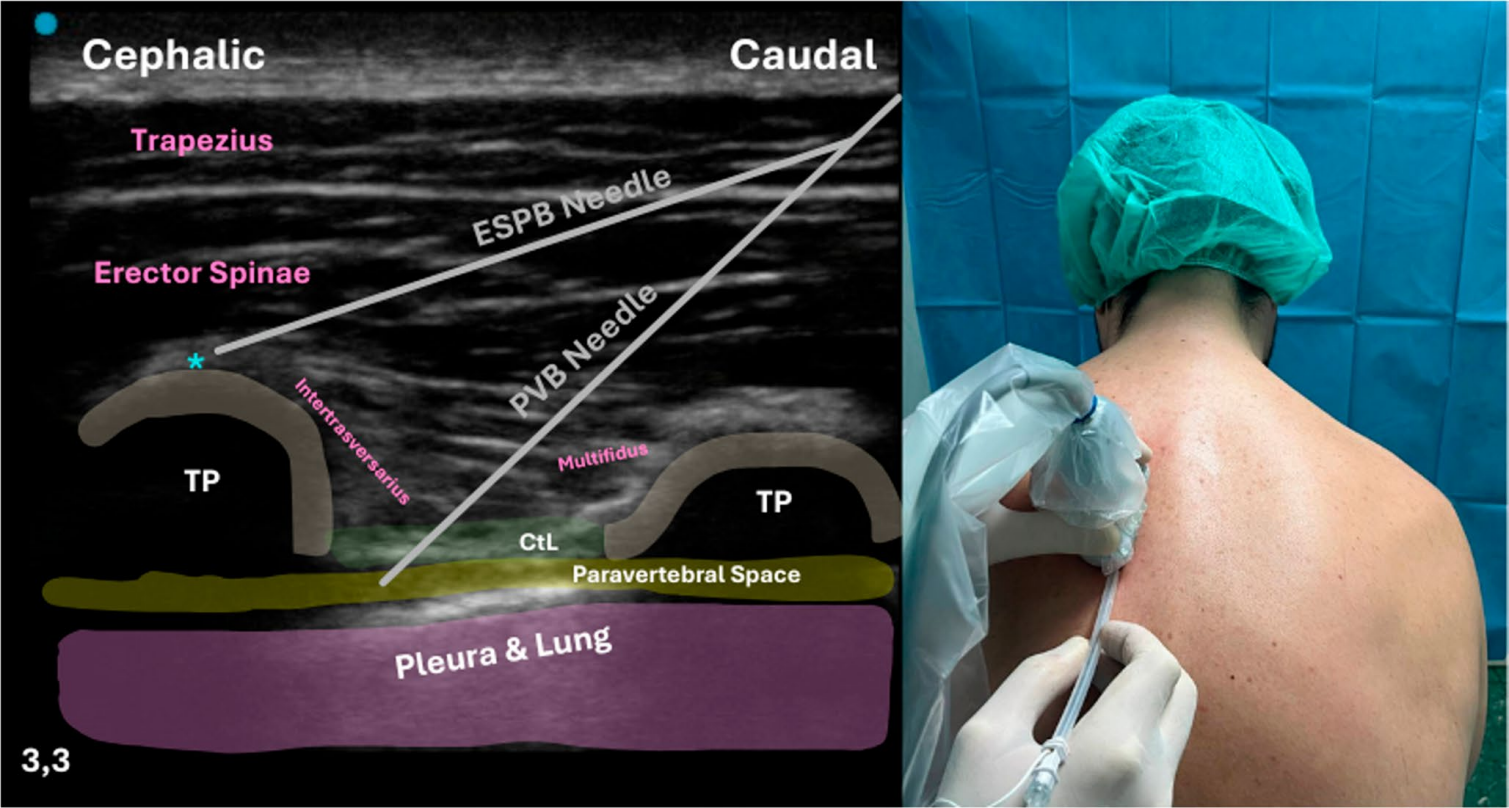
Ultrasound-guided ESPB block and PVB at the thoracic level. The schematic image on the left illustrates both techniques, which share similar sonoanatomical landmarks. The erector spinae plane block is performed by advancing the needle in-plane in a caudo-cranial direction until the tip reaches the fascial plane between the transverse process (TP) and the erector spinae muscle. The paravertebral block is performed deeper, with the needle directed toward the paravertebral space. On the right, the in-plane caudo-cranial needle approach is demonstrated on a patient; for the paravertebral block, a slight medial tilt of the transducer is required to visualize the pleura and the paravertebral compartment. Abbreviations: TP – Transverse Process; CtL – Costotransverse Ligament; ESPB – Erector Spinae Plane Block; PVB – Paravertebral Block

ESPB injecting local anesthetic deep to the erector spinae muscle at the thoracic level (typically T2-T3 for shoulder and upper arm procedures). Provides multisegmental sensory blockade by diffusing anteriorly to affect the dorsal and ventral rami of spinal nerves (Fig. 15) [70, 71]. In the context of PHFs, a recent narrative review by Fusco et al. (2025) summarized the available evidence—largely limited to case reports and small case series—suggesting that ESPB may provide effective postoperative analgesia while preserving diaphragmatic function, supporting its role as a phrenic nerve–sparing technique in selected patients [72]. Consistently, Diwan et al. (2020) demonstrated satisfactory analgesia for PHF surgery with ESPB and no clinically relevant respiratory impairment, highlighting its potential utility in patients at increased risk of phrenic nerve dysfunction [73].

PVB targets the paravertebral space, anesthetizing spinal nerve roots. Provides unilateral somatic and sympathetic blockade, reducing pain transmission from the shoulder and upper chest (Fig. 15). Potential benefits include effective intraoperative anesthesia and postoperative analgesia, reduced opioid requirements, and preservation of phrenic nerve function, making it particularly attractive in high-risk patients. Reported risks—such as pneumothorax, hypotension, or unintended epidural spread—are infrequent, especially with ultrasound guidance [74]. The feasibility of PVB as a primary anesthetic technique for shoulder surgery was described by Klein et al. (2004) in a case report, in which a continuous cervical PVB was successfully used as the sole anesthetic in an 85-year-old patient with severe cardiopulmonary disease who could not tolerate general anesthesia. The block provided excellent surgical conditions and effective postoperative analgesia, while allowing titrated dosing and continuous respiratory monitoring, supporting PVB as a viable alternative to ISBPB in selected patients requiring diaphragm-sparing strategies [75].

Diaphragm-sparing Techniques are summarized in Table 5.

**Table 5:** Summary of Diaphragm-sparing Techniques

Technique	Coverage	Advantages	Disadvantages
SSNB and AxNB	Shoulder joint	Opioid-sparing	Limited to shoulder, not effective for distal limb (SSNB) Does not cover whole upper limb (AxNB)
STB	C5-C6 roots	Reduced phrenic nerve involvement, effective analgesia	Limited to upper arm and shoulder
WALANT	Surgical field only	No general anesthesia, minimal systemic complications	Not suitable for all procedures and for all patients
ESP/PVB Blocks	Shoulder, thoracic region	Phrenic nerve-sparing, potential for prolonged analgesia	Requires expertise, limited evidence for some applications

Abbreviations: ASM – Anterior Scalene Muscle; MSM – Middle Scalene Muscle

Abbreviations: ST – Superior Trunk; ASM – Anterior Scalene Muscle; MSM – Middle Scalene Muscle

### Identification of Patients at Risk and Ultrasound Evaluation of the Diaphragm

The risk of phrenic nerve involvement during interscalene or supraclavicular blocks is not uniform across all patients. Individuals with chronic Obstructive Pulmonary Disease (COPD), Obstructive Sleep Apnea (OSA), obesity, or limited cardiopulmonary reserve are more susceptible to clinically relevant hemidiaphragmatic paralysis. Recognizing these patients preoperatively is essential to tailor the anesthetic plan and minimize respiratory complications [76].

Ultrasound assessment has become an invaluable tool not only for guiding RA but also for evaluating diaphragmatic function before and after block performance.

Through M-mode and B-mode ultrasound, clinicians can quantify diaphragmatic excursion, thickness, and thickening fraction, providing objective parameters to detect subclinical paresis and monitor recovery. These techniques are particularly useful at the bedside, allowing dynamic evaluation in real time [77].

Several studies have underlined the importance of needle positioning and local anesthetic spread in reducing phrenic nerve involvement.

Albrecht et al. (2017) demonstrated that minimizing local anesthetic volume and avoiding intrafascial spread around the BP sheath significantly decreases the incidence of hemidiaphragmatic paralysis [76].

Similarly, Ayyanagouda et al. (2019) confirmed that the extrafascial approach to ISBPB provides comparable analgesia with a markedly lower rate of diaphragmatic dysfunction [77].

More recently, Coviello et al. (2025) employed ultrasound to evaluate diaphragmatic motion and thickening fraction before and after ISBPB, finding that the extrafascial approach was associated with fewer hemodynamic variations and may therefore represent a safer option in patients with compromised pulmonary function [78].

Taken together, these findings highlight the importance of integrating pre-procedural risk stratification and ultrasound-based diaphragm monitoring into clinical practice. Such an approach allows anesthesiologists to select the most appropriate regional technique—balancing efficacy, safety, and patient-specific respiratory considerations.

### CP Block

Performing PHF surgery without general anesthesia requires more than BP block alone [79]. Although BP techniques provide effective anesthesia of the shoulder joint and proximal humerus, they do not consistently cover all sensory territories involved in surgical exposure and perioperative pain. For this reason, complete RA for PHF surgery often necessitates integration of a CP block [80]. This anatomical and functional interdependence explains why CP block cannot be considered separately from BP anatomy and technique when planning fully regional approaches.

#### Anatomical basis

The CP is formed by the anterior rami of the C1–C4 spinal nerves and lies deep to the SCM and anterior to the scalene muscles [81]. From a clinical perspective, its superficial sensory branches arising mainly from C3–C4 are the most relevant for shoulder and proximal humerus surgery. These branches emerge together at the posterior border of the SCM at the level of the C3–C4 vertebrae and provide cutaneous innervation to the clavicular region, superior shoulder, and deltoid area [81]. Although they do not directly innervate the glenohumeral joint capsule, they represent a major source of superficial surgical pain and therefore require specific blockade when PHFs surgery is performed without general anesthesia [82].

The efficacy and spread of CP blocks are strongly influenced by the layered anatomy of the cervical fascia [83, 84]. The superficial cervical fascia contains subcutaneous tissue and the platysma muscle, while the deep cervical fascia is classically divided into an investing, pretracheal, and prevertebral layer [84]. The investing layer encloses the SCM and trapezius muscles and constitutes the main anatomical reference for superficial and intermediate CP blocks [84, 85]. In contrast, the prevertebral fascia covers the scalene muscles and vertebral structures, separating the superficial CP from deeper neural elements, including the cervical nerve roots and the BP [84]. A clear understanding of these fascial planes is essential to determine the appropriate depth of injection, optimize cutaneous sensory coverage, and minimize unintended spread to deeper or medial structures.

#### Technical Considerations

The introduction of ultrasound guidance has substantially improved the accuracy and safety of CP blocks by enabling real-time visualization of relevant anatomical structures, needle advancement, and local anesthetic spread. This has significantly reduced the risk of vascular puncture, intraneural injection, and unintended deep or medial diffusion, making CP block a reproducible and clinically reliable adjunct in shoulder and proximal humerus surgery [80, 81].

**Fig. 16:**
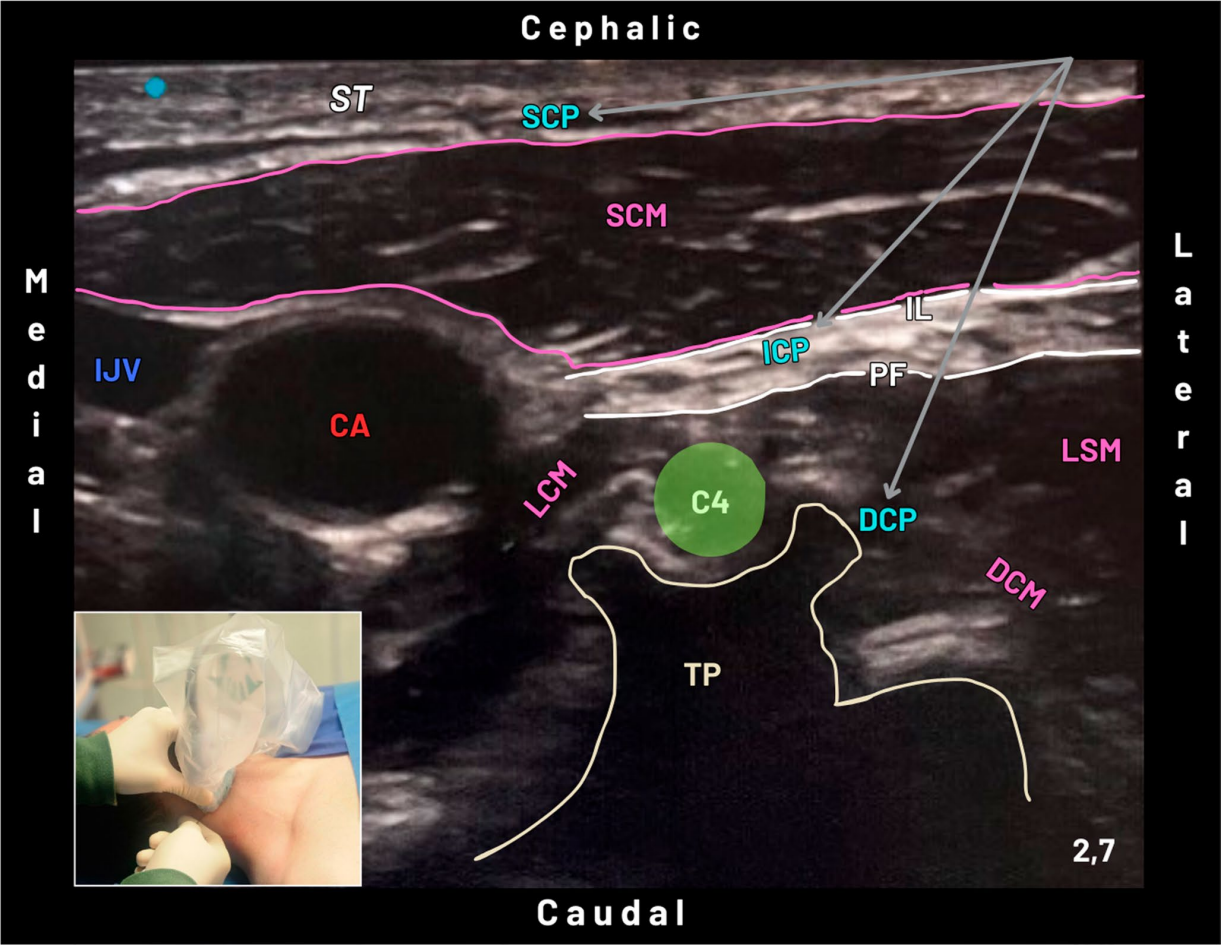
Ultrasound image at the level of the C4 transverse process showing the anatomical targets for Superficial (SCP), Intermediate (ICP), and Deep (DCP) Cervical Plexus Blocks. The arrows indicate the needle trajectory and the respective injection planes. The superficial cervical plexus block (SCP) targets the subcutaneous tissue (ST) along the posterior border of the sternocleidomastoid muscle (SCM). The intermediate cervical plexus block (ICP) involves deposition of local anesthetic within the fascial plane between the SCM (IL) and the prevertebral fascia (PF), corresponding to the deep layer of the deep cervical fascia, targeting the cervical plexus branches before their superficial emergence. The intermediate cervical plexus block (ICP) consists of local anesthetic deposition deep to the prevertebral fascia (PF), between this layer and the transverse process (TP), adjacent to the deep cervical muscles (DCM). In contrast to the superficial and intermediate techniques, the deep approach is associated with a less favorable safety profile. The inset (lower left) shows ultrasound-guided block performance using an in-plane lateral-to-medial needle approach. Abbreviations: CA - Carotid Artery; IJV - Internal Jugular Vein; TP – Transverse Process; C4 - Fourth Cervical Nerve Root; SCM - Sternocleidomastoid Muscle; LCM - Longus Capitis Muscle; DCM - Deep Cervical Muscles; LSM - Levator Scapulae Muscle; ST - Subcutaneous Tissue; SCP - Superficial Cervical Plexus Block; ICP - Intermediate Cervical Plexus Block; DCP - Deep Cervical Plexus Block; IL - Investing Layer (The Superficial Layer of the Deep Cervical Fascia); PF - Prevertebral Fascia

Several ultrasound-guided approaches to CP have been described, differing in depth of injection, extent of sensory coverage, and safety profile [86] (Fig. 16). The most superficial technique, referred to as the “Superficial CP Block”, targets the terminal sensory branches as they emerge at the posterior border of the SCM muscle, typically at its midpoint (Erb’s point). With the patient in a supine position and the head rotated contralaterally, a high-frequency linear probe is placed transversely over the lateral neck to identify the SCM, internal jugular vein, carotid artery, and underlying fascial planes. Local anesthetic is injected subcutaneously or immediately deep to the investing cervical fascia along the posterior border of the SCM [87]. This approach provides reliable cutaneous analgesia of the anterolateral neck and supraclavicular region with a very low risk of phrenic nerve involvement or motor block; however, its effect is limited to superficial structures and is therefore insufficient as a stand-alone technique for PHFs surgery [87, 88].

A deeper but still safe option is the “Intermediate CP Block”, which involves deposition of local anesthetic in the fascial plane between the SCM and the prevertebral fascia, targeting the CP branches before their superficial emergence [86]. Compared with the superficial approach, it offers a denser and more consistent sensory block while maintaining a favorable safety profile [89]. Under ultrasound guidance, the SCM, prevertebral fascia, and adjacent vascular structures can be clearly identified, allowing accurate injection of relatively low volumes of local anesthetic (typically 5–10 mL) [88, 90]. Clinically, this technique has been associated with improved analgesic efficacy compared with Superficial CP block, while still minimizing the risk of phrenic nerve block and deep cervical complications, and represents a balanced option when CP block is used as an adjunct to BP techniques [88–90].

In contrast, the “Deep CP Block” targets the cervical nerve roots (C2–C4) at the level of the transverse processes and, although capable of providing extensive sensory coverage, is associated with a significantly higher incidence of adverse events, including intravascular injection, epidural or intrathecal spread, phrenic nerve palsy, and recurrent laryngeal nerve block [86]. Even with ultrasound guidance, this approach remains technically demanding and presents an unfavorable risk–benefit profile, particularly in elderly or frail patients [91].

A more selective strategy, commonly referred to as “Selective CP Block”, focuses on blocking the supraclavicular nerves (C3–C4) specifically, using small volumes of local anesthetic deposited lateral to the SCM and superficial to the prevertebral fascia [92]. This technique aims to provide focused analgesia of the clavicular and superior shoulder region while avoiding unnecessary spread to deeper structures [92]. This targeted approach is particularly valuable as an adjunct to diaphragm-sparing BP techniques, such as STB or infraclavicular block, where it complements articular and periosteal anesthesia with effective coverage of superficial surgical territories [92, 93].

Overall, ultrasound-guided CP blocks should be regarded as complementary rather than stand-alone techniques for PHFs surgery. Their primary role is to enhance superficial analgesia, reduce perioperative opioid requirements, and improve patient comfort, especially when integrated into multimodal regional anesthesia strategies in combination with brachial plexus or fascial plane blocks.

## Expert perspectives and practical recommendations

### Choice of RA technique

The selection of the optimal RA technique for PHF surgery must account for various clinical factors, including the surgical procedure, patient respiratory function, comorbidities, and the anticipated need for prolonged postoperative analgesia [6].

Based on clinical experience, several considerations can guide anesthetic planning:

- In young, healthy, and active patients undergoing ORIF, the ISBPB remains a widely used reference technique, providing reliable coverage of the C5–C6 roots and excellent intraoperative conditions. ISBPB has been associated with superior functional outcomes and range of motion [6, 39–40]. To prolong analgesia and mitigate rebound pain, we recommend adjunctive use of intravenous dexamethasone and/or dexmedetomidine. Alternatively, the STB is a viable option when diaphragmatic function must be preserved. Comprehensive analgesia should be maintained throughout the early postoperative period to enable effective rehabilitation. In such cases, particularly for patients requiring rapid functional recovery, perineural catheters are recommended. Infraclavicular blocks are increasingly preferred for continuous catheter-based analgesia due to their deeper anatomical location, which allows stable catheter placement and minimizes displacement.
- In emergency settings, particularly in elderly patients with significant cardiopulmonary compromise, RA may facilitate surgical intervention while avoiding general anesthesia.
- In patients with underlying pulmonary conditions (e.g., COPD, OSA, obesity and hypoventilation syndrome), techniques that spare the phrenic nerve should be prioritized. For such high-risk patients, the STB offers analgesia comparable to ISBPB with a significantly lower incidence of hemidiaphragmatic paresis. This block is particularly suitable for procedures involving the glenohumeral joint, with minimal motor impairment of the distal upper limb. In more extensive surgeries, such as Reverse Shoulder Arthroplasty (RSA), a combination SSNB + AxNB may selectively anesthetize the shoulder while preserving diaphragmatic and limb motor function. In frail or anticoagulated patients in whom deeper plexus blocks are contraindicated, the ESPB offers a safer alternative, providing effective unilateral analgesia with a reduced risk of complications. Continuous Peripheral Nerve Block (CPNB) with secure catheter positioning may ensure adequate analgesia during the critical first 48–72 postoperative hours, supporting early rehabilitation. Ultimately, a patient-centered, context-specific approach is essential. No single technique is universally superior; rather, RA should be tailored to optimize safety, analgesia, and surgical outcomes in each PHF case.

### Tips and technical considerations

The efficacy and safety of RA for PHFs depend not only on anatomical knowledge but also on precise technical refinements. Several aspects—including ultrasound technique, needle selection, injection pressure monitoring, and local anesthetic dosing—can significantly influence outcomes.

#### Ultrasound and needle technique

High-frequency linear probes (6–15 MHz) are preferred for most superficial approaches, such as interscalene, supraclavicular, and superior trunk blocks. Curvilinear low-frequency probes (2–5 MHz) are rarely required, even in obese patients, since the target structures are generally superficial; however, they may improve visualization in deeper regions, such as the infraclavicular or paravertebral areas. The use of linear probes also allows optimal visualization of the needle along its entire trajectory. In-plane needle advancement enables continuous visualization of both the needle shaft and tip, thereby increasing procedural safety—particularly when working near major vessels or other critical structures, such as the pleura. However, during ISBPB, the in-plane approach typically requires needle passage through the MS muscle. In this region, the dorsal scapular nerve and the long thoracic nerve commonly course within or through the muscle and may therefore be at risk of mechanical injury. As these nerves are predominantly motor, injury may occur without reliable paresthesia warning and can result in clinically relevant shoulder girdle dysfunction. As an alternative, an out-of-plane needle approach may be considered ISBPB, as it allows the needle trajectory to avoid traversing the MS muscle, potentially reducing the risk of injury to the dorsal scapular and long thoracic nerves [94]. Out-of-plane techniques may be employed when space is limited or for catheter placement, provided that hydrodissection confirms appropriate local anesthetic spread. The ESPB and the infraclavicular block are the most frequently described techniques performed with an out-of-plane approach [45, 71, 73]. However, in our opinion, these approaches—though seemingly simpler—should be reserved for operators who are thoroughly familiar with the sonoanatomy and the potential structures the needle may encounter along its path.

A detailed understanding of sonoanatomy is essential in this context to minimize the risk of injury. Echogenic, short-bevel, insulated needles (21–22 G, 50–100 mm) are recommended for optimal sonographic visibility and tactile feedback.

#### Injection pressure and dual guidance

Continuous monitoring of injection pressure, combined with dual guidance (ultrasound and nerve stimulation), can reduce the risk of intraneural injection. Safe injection pressure should always be ensured using dedicated monitoring systems, maintaining values below 15 Pound Per Square Inch (PSI) and confirming smooth, low-resistance injection. Nerve stimulation (0.3–0.5 mA) may assist in identifying motor responses (e.g., deltoid or biceps contraction) and verifying needle proximity before injection. However, its use may not always be feasible in patients with fractures, as it can provoke pain or unwanted muscle contractions.

#### Local anesthetic selection and adjuvants

Ropivacaine and Levobupivacaine (0.25–0.5%) are commonly employed for single-injection techniques, providing 8–14 h of analgesia [95–97].For prolonged effect, perineural or intravenous Dexamethasone (4–8 mg) and Dexmedetomidine (0.5–1 µg/kg) can be used as adjuvants [98, 99]. Randomized controlled trials have demonstrated that perineural Dexamethasone significantly decreases rebound pain and opioid consumption within the first 48 h postoperatively [100, 101]. In contrast, the use of mixtures of short- and long-acting local anesthetics is no longer recommended, as recent evidence indicates that such combinations do not reliably confer the expected advantages of rapid onset and prolonged duration, and may result in unpredictable pharmacodynamic profiles without clear clinical benefit. When specific onset or duration characteristics are desired, optimization of local anesthetic concentration and volume, together with the judicious use of adjuvants, should be preferred over anesthetic mixtures [102, 103].

#### Approach-specific tips

##### ISBPB

Perform “trace-back” scanning to identify C5–C6 roots and their relationship to the scalene muscles [78]. Use minimal effective volume (5–10 mL) and extrafascial injection to limit spread to other structures (the phrenic nerve) [78].

##### Supraclavicular Block

Target the “corner pocket” between the inferior trunk and first rib [43]. Maintain shallow needle angle and use Doppler to avoid the subclavian vessels. In the supraclavicular approach, ligamentous and connective tissue structures between the superior and inferior trunks may act as anatomical barriers, potentially limiting local anesthetic spread and affecting the extent and consistency of analgesia. To mitigate this ‘barrier effect’, a single skin puncture with two careful needle redirections—targeting both the superior and inferior trunk compartments—may facilitate a more homogeneous distribution of local anesthetic and improve block coverage.

##### Infraclavicular Block

For PHFs, arm abduction is often limited or contraindicated due to pain or instability [45]. In these cases, the costoclavicular approach is preferred, as it can be performed with the arm in a neutral position. If tolerated, gentle abduction (≤ 30°) may slightly improve visualization of the cords without exacerbating fracture displacement. Catheter placement should follow the posterior aspect of the axillary artery within the clavipectoral fascia to enhance stability.

##### STB

The injection should be performed lateral to the C5–C6 convergence to avoid involvement of the phrenic nerve, ensuring that the local anesthetic spreads between the AS and MS muscles. Particular attention should be paid to the suprascapular nerve, which branches from the BP immediately after its division into trunks. Achieving blockade of this nerve is crucial to provide effective analgesic coverage of the shoulder [66].

##### SSNB and AxNB

Employ small, targeted volumes (3–5 mL per site) using an in-plane posterior approach for the suprascapular nerve and a posterior quadrilateral space approach for the AN [63]. In specific patient populations—such as those with obesity, altered anatomy, or anticoagulation—lower-frequency probes and hydrodissection with saline can improve needle visualization and safety. In these high-risk cases, fascial plane blocks such as ESPB may represent safer alternatives. Ultimately, expert-level performance requires balancing anatomical precision with adaptability to individual patient characteristics. The combined use of real-time ultrasound imaging, injection pressure monitoring, and evidence-based local anesthetic dosing remains the cornerstone of safe and effective RA practice.

##### CP blocks

Ultrasound-guided CP block should be considered a valuable adjunct rather than a primary anesthetic technique in PHFs surgery. Among the available approaches, the “Intermediate CP Block” and Selective block of the Superficial Branches provide the most favorable balance between analgesic efficacy and safety [88–90].

In our practice, the Intermediate ultrasound-guided approach is preferred, as it offers more consistent sensory coverage of the supraclavicular and clavicular regions compared with superficial injection, while maintaining a low risk of phrenic nerve involvement and deep cervical complications. This is particularly relevant in elderly patients or those with impaired respiratory reserve, in whom preservation of diaphragmatic function is a priority.

When combined with diaphragm-sparing BP techniques—such as STB or infraclavicular block—CP block enhances overall analgesic coverage of the shoulder girdle by addressing cutaneous territories frequently spared by BP anesthesia alone. This integrated regional strategy may reduce the need for intraoperative supplementation, improve postoperative pain control, and limit opioid consumption. Conversely, we do not routinely recommend “Deep CP Block” for PHFs surgery, even under ultrasound guidance, because of its higher complication profile and the limited incremental benefit compared with safer, more selective techniques.

### Postoperative analgesia: continuous catheter techniques versus single-injection blocks

Optimal postoperative pain management is critical for functional recovery following PHFs surgery. Two commonly used strategies include single-injection nerve blocks with adjuvants and CPNB via perineural catheters [4, 6].

#### Single-Injection Blocks

ISBPB remains widely used due to its simplicity, rapid onset, and reliable analgesic efficacy for PHFs surgery [104]. However, its main limitation lies in the finite duration of analgesia and the potential for rebound pain once the block resolves [12, 105]. While single-injection blocks provide excellent intraoperative conditions and early postoperative comfort, their short-lived effect may be inadequate for patients requiring prolonged pain control or delayed mobilization. In such cases, continuous catheter techniques or multimodal analgesic strategies should be considered to ensure sustained pain relief and facilitate rehabilitation [105]. From an expert standpoint, single-injection techniques are most appropriate for low-risk patients undergoing shorter procedures, where simplicity, speed, and avoidance of catheter-related complications are prioritized. Conversely, for complex or prolonged surgeries, their role is best viewed as part of a broader multimodal analgesic approach rather than a stand-alone strategy.

#### CPNB

CPNB provide sustained analgesia through continuous local anesthetic infusion, improving pain control, reducing opioid use, and enhancing rehabilitation [106]. They are associated with fewer opioid-related side effects, better sleep quality, and higher patient satisfaction [106, 107]. However, proper catheter management is essential to avoid complications such as dislodgement or infection [106, 107].

**Catheter Placement Approaches**:

- **ISBPB**: Traditionally used for PHFs surgery due to reliable C5–C6 blockade, but associated with a high incidence of hemidiaphragmatic paralysis—nearly 100%—posing concerns in patients with limited pulmonary reserve [5].
- **STB**: Targets the upper trunk with lower risk of phrenic nerve palsy. One study reported hemidiaphragmatic paresis in 72.5% of ISBPB recipients versus 5.3% in those receiving STB [108].
- **Supraclavicular Block**: Offers dense BP anesthesia and is associated with a lower incidence of diaphragmatic paralysis compared with ISBPB, while ensuring effective intraoperative anesthesia and postoperative analgesia for upper limb procedures [109]. The principal risk, particularly with continuous supraclavicular block, is pneumothorax, owing to the close proximity of the pleura to the BP at the supraclavicular level. Although the use of real-time ultrasound guidance has markedly reduced the incidence of clinically significant pneumothorax to approximately 0.06% in large series, the risk is not eliminated and may increase with suboptimal needle visualization or limited operator experience, warranting careful patient selection and meticulous technique [110].
- **Infraclavicular Block (Costoclavicular Approach)**: The costoclavicular approach represents a reliable and diaphragm-sparing alternative for continuous BP anesthesia in PHFs surgery. The block is performed with the patient in a supine or slight head-up position, and arm abduction is not required—a key advantage in patients with restricted mobility or unstable fractures. A high-frequency linear transducer is placed immediately below the midclavicle to identify the three cords of the brachial plexus clustered lateral to the axillary artery within the costoclavicular space. The needle is advanced in-plane from lateral to medial, and local anesthetic is deposited within the clavipectoral fascia, ensuring uniform spread around all cords. For catheter placement, a 19–20 G multi-orifice catheter is inserted 3–4 cm beyond the needle tip to optimize stability and drug distribution. Catheters are typically positioned postoperatively, after surgical closure, and tunneled laterally to maintain the entry point outside the sterile field. Fixation is achieved using adhesive securing devices and tissue glue, followed by transparent occlusive dressing to reduce infection risk. Postoperative analgesia is maintained via elastomeric or electronic pump systems, delivering ropivacaine 0.2% at 5–7 mL/h with optional patient-controlled boluses (2–3 mL every 30–60 min). In our institution, continuous infusions are usually maintained for 48–72 h, after which the catheter is removed in the ward setting. Selected patients with good compliance and home support may be discharged with a portable pump system under remote supervision by the acute pain team. Recent clinical data support the efficacy and safety of this technique [111–115]. When continuous infusion is not feasible, single-shot blocks using liposomal bupivacaine can provide extended analgesia while avoiding the complexity of catheter management [116].

Overall, clinical studies indicate that continuous infraclavicular block approach offer effective analgesia with a favorable safety profile. For instance, randomized trials have shown fewer postoperative sleep disturbances with infraclavicular catheters compared to supraclavicular ones [117].

In conclusion, although ISBPB remains a well-established option, ST and infraclavicular approaches provide comparable analgesia with a lower risk of respiratory complications. The choice of technique should be individualized based on patient comorbidities, surgical extent, and safety considerations.

## Study limitations

This review has several limitations. First, it was conducted as a narrative review supported by a structured but non-systematic literature search, which may have led to incomplete retrieval of relevant studies despite using multiple databases. Second, the number of eligible clinical investigations specifically addressing RA for PHFs surgery was limited, restricting the strength and generalizability of the conclusions. Third, the included studies were heterogeneous in terms of design, sample size, anesthetic techniques, and outcome measures, precluding any formal meta-analysis and limiting comparability. Fourth, additional references were included for anatomical, surgical, and clinical context but were not part of the formal synthesis; while valuable for interpretation, these sources cannot replace high-quality clinical evidence. Finally, our “Expert Perspectives” section reflects real-world experience rather than formal consensus methodology, which, although clinically relevant, may introduce subjectivity.

## Conclusions

RA plays a fundamental role in the surgical management of PHFs, providing effective analgesia, reducing opioid consumption, and, in selected patients, allowing the avoidance of general anesthesia. However, the PHF-specific evidence base remains limited and heterogeneous, as most available studies are small, observational, and focused primarily on short-term analgesic outcomes and respiratory safety rather than functional recovery or long-term results. Within these constraints, current data support the feasibility of several RA techniques in this clinical setting.

Beyond the included studies, additional anatomical, surgical, and anesthetic literature provides important context for interpreting these findings and for guiding clinical practice. This broader body of evidence highlights how fracture pattern, surgical approach, and patient-specific factors—particularly cardiopulmonary reserve and comorbidities—should inform the choice of regional technique. In this framework, ISBPB remains an effective option for carefully selected patients, while diaphragm-sparing strategies, such as STB, infraclavicular approaches, or combined suprascapular and axillary nerve blocks, offer rational alternatives in individuals at increased risk of respiratory compromise.

Integrating available evidence with multidisciplinary expert experience, continuous peripheral nerve blocks may be considered to extend postoperative analgesia, reduce opioid requirements, and facilitate early mobilization, especially in patients in whom prolonged pain control is critical to rehabilitation. Conversely, more extensive blocks should be reserved for carefully selected individuals in whom the expected benefits outweigh potential respiratory or neurological risks. Overall, RA for PHF surgery should be approached as an individualized, patient-centered process that balances analgesic efficacy, safety, and functional recovery.

Despite growing clinical interest, robust high-quality evidence specifically addressing RA in PHF surgery is still lacking. Future well-designed prospective studies and randomized controlled trials are needed to strengthen the evidence base, refine patient selection criteria, optimize technique choice, and evaluate functional, respiratory, and long-term outcomes. In the meantime, a tailored, multidisciplinary approach that integrates clinical evidence, anatomical and surgical considerations, and expert judgment remains essential to ensure safe and effective perioperative care for patients with PHFs.

## Data Availability

No datasets were generated or analysed during the current study.

## Abbreviations

AA: Axillary Artery
AN: Axillary Nerve
AS: Anterior Scalene (muscle)
AV: Axillary Vein
AVN: Avascular Necrosis
AxNB: Axillary Nerve Block
BP: Brachial Plexus
C5, C6, etc.: Cervical Nerve Root 5, 6, etc
COPD: Obstructive Pulmonary Disease
CP: Cervical Plexus
CPNB: Continuous Peripheral Nerve Block
ESPB: Erector Spinae Plane Block
GT: Greater Tuberosity
HA: Hemiarthroplasty
HH: Humeral Head
ICBN: Intercostobrachial nerve
IMN: Intramedullary Nailing
ISBPB: Interscalene Brachial Plexus Block
LC, MC, PC: Lateral Cord, Medial Cord, Posterior Cord (of the Brachial Plexus)
LT: Lesser Tuberosity
MS: Middle Scalene (muscle)
ORIF: Open Reduction and Internal Fixation
OSA: Obstructive Sleep Apnea
PSI: Pound Per Square Inch
PVB: Paravertebral Block
PHFs: Proximal Humerus Fractures
Pm: Pectoralis Minor
PM: Pectoralis Major
RA: Regional Anesthesia
RAPTIR: Retroclavicular Approach to the Infraclavicular Region
RSA: Reverse Shoulder Arthroplasty
SA: Subclavian Artery
SCM: Sternocleidomastoid Muscle
SN: Suprascapular Nerve
SSNB: Suprascapular Nerve Block
ST: Superior Trunk (of the Brachial Plexus)
STB: Superior Trunk Block
WALANT: Wide Awake Local Anesthesia No Tourniquet

## Declarations

**Clinical Trial Number**.
